# Downstream Allosteric Modulation of NMDA Receptors by 3-Benzazepine Derivatives

**DOI:** 10.1007/s12035-023-03526-1

**Published:** 2023-08-05

**Authors:** Nadine Ritter, Paul Disse, Isabel Aymanns, Lena Mücher, Julian A. Schreiber, Christoph Brenker, Timo Strünker, Dirk Schepmann, Thomas Budde, Nathalie Strutz-Seebohm, Simon M. Ametamey, Bernhard Wünsch, Guiscard Seebohm

**Affiliations:** 1 Institute for Genetics of Heart Diseases (IfGH), Department of Cardiovascular Medicine, University Hospital Münster, 48149 Münster, Germany; 2 Chembion, University of Münster, 48149 Münster, Germany; 3 Institute of Pharmaceutical and Medicinal Chemistry, University of Münster, Corrensstr. 48, 48149 Münster, Germany; 4 Centre of Reproductive Medicine and Andrology, University Hospital Münster, University of Münster, Domagkstr. 11, 48149 Münster, Germany; 5 Institute of Physiology I, University of Münster, 48149 Münster, Germany; 6 Institute of Pharmaceutical Sciences, ETH Zürich, Zürich, Switzerland

**Keywords:** iGluRs, GluN2B, Ifenprodil, Alternative splicing, 3-Benzazepine

## Abstract

*N*-Methyl-D-aspartate receptors (NMDARs) composed of different splice variants display distinct pH sensitivities and are crucial for learning and memory, as well as for inflammatory or injury processes. Dysregulation of the NMDAR has been linked to diseases like Parkinson’s, Alzheimer’s, schizophrenia, and drug addiction. The development of selective receptor modulators, therefore, constitutes a promising approach for numerous therapeutical applications. Here, we identified *(R)-*OF-NB1 as a promising splice variant selective NMDAR antagonist. We investigated the interaction of (*R*)-OF-NB1 and NMDAR from a biochemical, bioinformatical, and electrophysiological perspective to characterize the downstream allosteric modulation of NMDAR by 3-benzazepine derivatives. The allosteric modulatory pathway starts at the ifenprodil binding pocket in the amino terminal domain and immobilizes the connecting α5-helix to the ligand binding domain, resulting in inhibition. In contrast, the exon 5 splice variant GluN1-1b elevates the NMDARs flexibility and promotes the open state of its ligand binding domain.

**Supplementary Information:**

The online version contains supplementary material available at 10.1007/s12035-023-03526-1.

## Introduction

In the central nervous system (CNS), NMDARs are located postsynaptically and are involved in several processes in the CNS like learning, memory formation, long-term potentiation (LTP), synaptic plasticity, and stimuli conductance [1–5]. Overactivation of NMDARs leads to Ca^2+^-induced excitotoxicity, which has important implications for many neurological disorders, including stroke, Parkinson’s, Alzheimer’s, schizophrenia, and drug addiction [6–9]. NMDARs are therefore promising pharmacological targets for negative allosteric modulators [2, 5, 10]. Under resting conditions, Mg^2+^ blocks the NMDAR's pore, which is only removed upon membrane depolarization. Release of Mg^2+^ block leads, together with simultaneous binding of glycine and (*S*)-glutamate to the ligand binding domain (LBD), to channel opening [5, 11–20].

Seven genes encode for NMDAR subunits: a single gene *grin1* for GluN1, four genes *grin2* for GluN2A-D, and two genes *grin3* for GluN3A-B [5]. Furthermore, there are eight splice variants of GluN1 (1-1a to 1-4b) that can differ in three alternatively spliced exons: exon 5, 21, and 22 [21]. Exon 5 consists of 21 amino acids and is located in the GluN1-1b, GluN1-2b, GluN1-3b, and GluN1-4b amino terminal domain (ATD). NMDARs containing exon 5 exert a decreased agonist potency [12]. Additionally, exon 5 provides an interdomain contact between the LBD of the GluN1 and GluN2 subunits and structurally connects the ATD and LBD. Exon 5 stabilizes the ATD-LBD and LBD-LBD interfaces and is thereby involved in reducing the proton sensitivity of the NMDAR [22, 23]. Exon 5 containing NMDARs show decreased sensitivity to ifenprodil as well as spermine [12]. While the presence of exon 5 increases the rate of NMDAR deactivation, it decreases the proton sensitivity of the receptor, and therefore, exon 5 is a molecular modulator module of the NMDAR [23, 24]. In rat myenteric plexus NMDARs, exon 5 is involved in colonic inflammation processes, i.e., colitis. In line, GluN1-1b carrying exon 5 was upregulated 14, 21, and 28 days after induced colitis. Since inflammation results in a local pH drop and exon 5 results in a decreased pH sensitivity, expressing exon 5 containing NMDARs may conserve the NMDAR activity during inflammation [25].

**Fig. 1:**
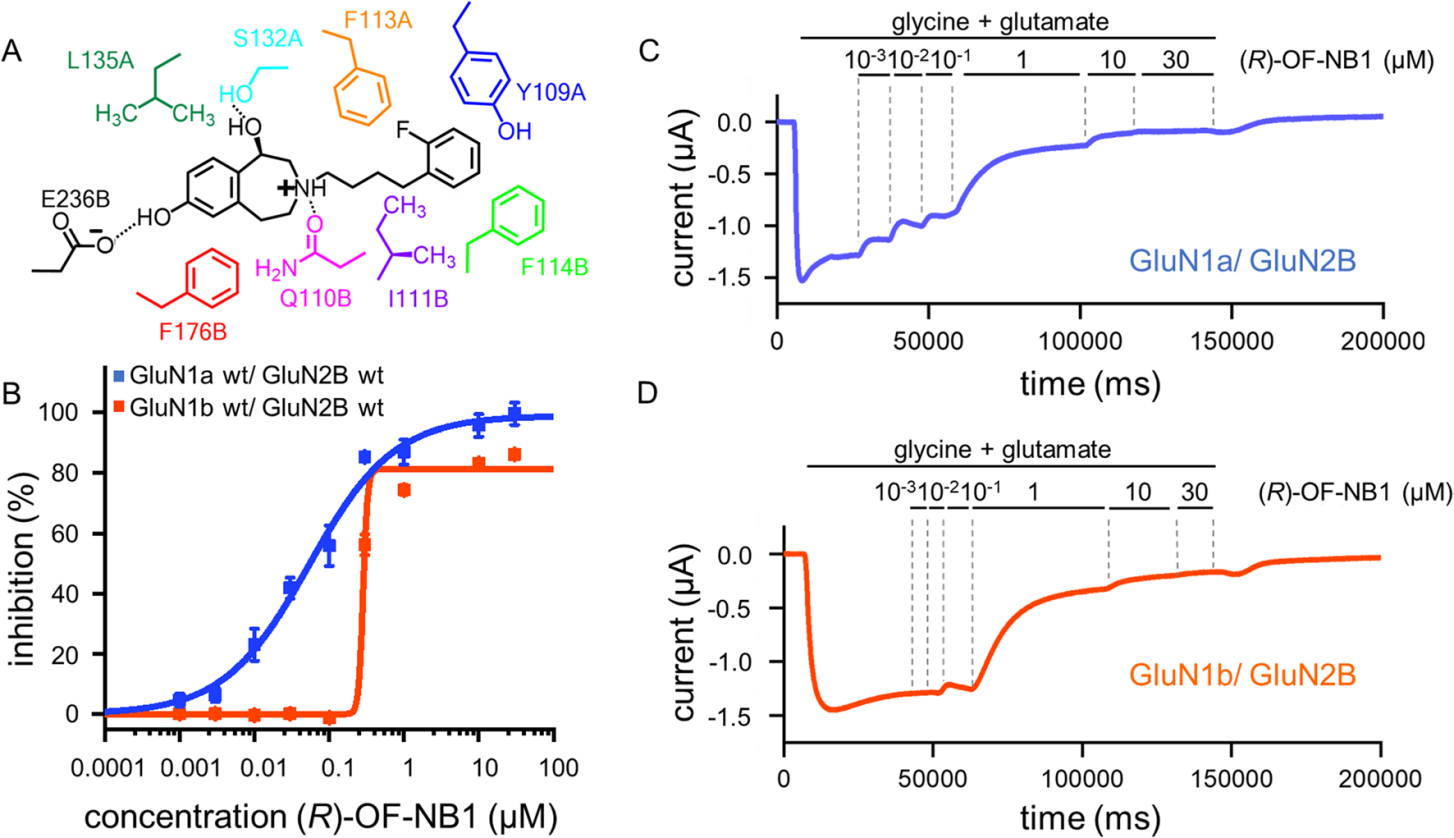
(*R*)-OF-NB1 in the ifenprodil binding pocket. The binding pocket is located at the interface between the GluN1 and GluN2B subunits within the ATD. Five interaction zones were previously described [28]. The respective amino acids in these interaction zones interact with the ligand via hydrophobic, aromatic, and H-bond interactions (**A**). Example traces of TEVC recordings obtained from GluN1 wt/ GluN2B wt expressing oocytes. NMDARs were first activated by applying 10 µM glycine and 10 µM (*S*)-glutamate and then inhibited by ascending (*R*)-OF-NB1 concentrations in presence of the two agonists (1, 3, 10, 30, 100, 300, 1000, 10,000, 30,000 nM). Oocytes were injected with 0.8 ng cRNA of each subunit and recorded after 5 days. Dose response curves were assessed by clamping the oocytes at a holding potential of − 70 mV. GluN1a/GluN2B expressing oocytes were already inhibited at lower antagonist concentrations (0.001 µM) (**C**) than GluN1b/2B expressing oocytes (~ 1 µM) (**D**). Dose response curves of (*R*)-OF-NB1 at GluN1b/GluN2B (orange) and GluN1a/GluN2B (blue), recorded by TEVC (**B**). The inhibition (%) was plotted against the concentration (µM). Curves were generated from recordings of *n* = 7–10 independent oocytes for each splice variant

Compounds like ifenprodil stabilize a non-conductive state of the NMDAR, block the spermine binding site, allosterically inhibit glycine binding, and stabilize the agonist binding at low open probability [23, 26, 27]. However, ifenprodil exerts low selectivity and binds to different α-adrenergic and serotonergic receptors [27]. Five interaction zones (IZ1-5) have been identified within the ifenprodil binding site in NMDARs [28]: Simultaneous interactions with IZ1, 2, 3, and 5 are crucial for potent inhibitory activity in GluN2B containing NMDARs. IZ1 is located at the GluN2B subunit and consists of E236, forming a H-bond between the deprotonated carboxy group and the phenolic group of the ligand (Fig. 1A). Hydrophobic and H-bond interactions with the GluN1 loop formed by residues GluN1-1a S132-L135 and the benzylic OH^−^ group are contributing to IZ2. IZ3 and IZ5 allow for aromatic interactions in the GluN2B subunit with amino acids F114 and F176. IZ4 exhibits hydrophobic interactions with GluN1-1a F113 and GluN2B I111, as well as an H-bond between the protonated tertiary amine of the tetrahydro-3-benzazepine scaffold, and the terminal carbamoyl group of GluN2B Q110 F176 is part of the α5-helix and was found to be important for the activation of the NMDAR. Upon receptor activation, F176 is placed vertically at the upper α5-helix and allows structural rearrangements. In the context of deactivation and/ or inhibition, F176 flips to a horizontal position. This flip is accompanied by a complete rearrangement of the GluN2 subunit and allows the formation of the accessible inhibitor binding pocket [28].

The α5-helix is located at the GluN2B ATD-LBD interface [29]. The LBD provides a direct route of modulation between the ATD and the pore via its clamshell closure rearrangement. The rearrangement of the ATD directly translates into the LBD [30]. Ifenprodil derivatives alter the contact between ATD and LBD. During binding, the α5-α6-loop movement is hindered, and the α5-α6-loop shows an extended distance to loop 2, which results in reduced interactions. Therefore, ifenprodil derivatives act like an α5-helix decoupler from the GluN1 LBD and promote channel closure via GluN1/ GluN2B LBD movement towards the pore [29]. According to Lü et al. [29], interactions between amino acid residues N192 (N187) and F194 (F189) (adapted to PDB structure 6CNA) at the end of α5-helix were also found to structurally connect ATD and LBD interfaces of GluN1 and GluN2B subunits. N192 shows H-bond interactions with R510 (R487) and polar interactions towards N515 (N429). Additionally, F194 shows cation-π interactions with R725 (R692) and R431 (R424) in silico.

Dysregulation of NMDARs by overactivation has been associated with excitotoxicity in a variety of diseases, including Parkinson’s, Alzheimer’s, schizophrenia, and drug addiction [6–9]. Therefore, the NMDAR displays a promising pharmacological target for negative allosteric modulators. The 3-benzazepine WMS-14–10 was derived from the NMDAR antagonist ifenprodil and was found to be a potent and selective GluN2B inhibitor that served as a start structure for the synthesis of (*R*)-OF-NB1 [31]. As various 3-benzazepines have already been shown to inhibit GluN2B activity in different biological systems, here we investigated the splice variant selectivity of (*R*)-OF-NB1 as a promising GluN2B-selective ifenprodil derivative [32]. In this context, we also investigated the general mechanism of negative allosteric modulation of NMDARs by 3-benzazepine derivatives, such as (*R*)-OF-NB1, both, in silico, and in vitro. In order to understand the modulation by 3-benzazepine derivatives, we investigated the allosteric route between starting from the ATD and extending to the LBD layers. We further aimed to investigate the importance of exon 5 for splice variant selectivity of (*R*)-OF-NB1.

## Material and Methods

### Molecular Biology

Single point mutations were incorporated into template DNA (GluN1-1b wt/ pSGEM and GluN2B wt/ pSGEM) by using the QuikChange II XL site-directed mutagenesis kit (Agilent Technologies, Germany). Mutagenesis primers were designed using the QuikChange Primer Design Program (Agilent) and provided by Microsynth (Microsynth AG, Balgach, Switzerland). For mutation of the desired amino acid in the gene, PCR amplification was performed with the respective mutagenesis primers (Eppendorf MasterCycler Gradient, Eppendorf Hamburg, Germany). Mutations were verified by automated DNA sequencing. Constructs were linearized with *PacI* (GluN1) or *NheI* (GluN2B). cRNA was prepared byin vitrotranscription using the mMessage mMachine T7 kit (Life Technologies, Darmstadt, Germany).

### RNA Injection in *Xenopus Laevis* Oocytes

The NMDAR was expressed in stage V and VI oocytes originated from *X. laevis*. Oocytes were provided by Ecocyte Bioscience (Dortmund, Germany), and equimolar amounts of wild-type and mutated GluN1 and GluN2B cRNA were injected (0.8 ng each). The oocytes were incubated in Barth’s solution, containing (mmol l^−1^): 88 NaCl, 1 KCl, 0.4 CaCl_2_, 0.33 Ca(NO_3_)_2_, 0.6 MgSO_4_, 5 TRIS–HCl, 2.4 NaHCO_3_, supplemented with 80 mg l^−1^ theophylline, 63 mg l^−1^ benzylpenicillin, 40 mg l^−1^ streptomycin, and 100 mg l^−1^ gentamycin and maintained at 18 °C until usage. After injection, the oocytes were incubated for 5 days at 18 °C. Subsequently, the activity of the expressed NMDARs in the oocytes was recorded by two-electrode voltage clamp (TEVC).

### Compound Solutions and TEVC Recordings

The compounds were retrieved as 10 mM stock solutions in DMSO and were subsequently diluted with agonist solution and adjusted to 0.1% DMSO concentration. Inhibition of each compound was measured via TEVC in *X. laevis* oocytes at room temperature using a Turbo Tec 10CX amplifier (NPI electronic, Tamm, Germany), NI USB 6221 DA/AD Interface (National Instruments, Austin, USA), and GePulse Software (Dr. Michael Pusch, Genova, Italy). Ba^2+^-Ringer, consisting of 10 mM HEPES, 90 mM NaCl, 1 mM KCl, and 1.5 mM BaCl_2_, adjusted to pH 7.4, was used for TEVC measurements. Agonist solution containing 10 µM glycine and 10 µM (*S*)-glutamate was prepared in Ba^2+^-Ringer, and the measurements were performed at a holding potential of − 70 mV, using electrodes with a glass capillary filled with 3 M KCl and a resistance between 0.5 and 1.5 MΩ. The compounds were tested by applying ascending concentrations, ranging from 1 to 30,000 nM, in at least six oocytes. Example traces for all recorded conditions can be found in Supplementary Figs. S2–S9.

### Data Analysis

TEVC data were analyzed using Ana (Dr. Michael Pusch, Genova, Italy), and statistics were performed using OriginPro 2020 (OriginLab Corporation, Northampton, USA). The inhibitory effect of each compound is calculated using the following equation:

$$\mathrm{inhibition}=1-\frac{{I}_{c}-{I}_{h}}{{I}_{a}-{I}_{h}}$$

where *I*_*h*_ describes the current in the absence of agonists; *I*_*a*_ represents the steady-state current in the presence of the agonists; and *I*_*c*_ is defined as the steady-state after binding of agonist and compound. All dose response curves are fitted to the following logistic equation:

$$\mathrm{y}=\frac{{A}_{1}-{A}_{2}}{1+{\left(\frac{\mathrm{x}}{{\mathrm{x}}_{0}}\right)}^{p}}+{A}_{2}$$

where *A*_*1*_ describes the minimal inhibition of a compound and was set to 0; *A*_*2*_ represents the maximal inhibition of a compound; *p* is the slope of the curve; *x*_*0*_ is defined as the concentration at half-maximal inhibition; and *x* is the tested concentration. IC_50_, A2, and hill slopes are listed in Table S1-3. Data were statistically analyzed by one-way ANOVA and Tukey post-hoc mean comparison test in OriginPro 2020 (OriginLab Corporation, Northampton, USA). *p* values are indicated by * for *p* < 0.05, ** for *p* < 0.01, and *** for *p* < 0.001.

### Voltage Clamp Recordings

Chinese hamster ovary (CHO) cells were co-transfected with GluN1-1a eGFP and GluN2B pDsRed2-N1 or GluN1-1b eGFP and GluN2B pDsRed2-N1. The constructs were a generous gift from Prof. Hollmann (Bochum, Germany). GluN1-1b eGFP was obtained by subcloning of GluN1-1b in eGFP using *BssHII* and *BtrI*. Single-channel patch clamp recordings were performed at room temperature using an Axopatch 200B amplifier (Molecular Devices, USA). The patch clamp experiments on cultured CHO cells were done using Clampex 10.7 software (Molecular Devices, USA). Single-channel recordings were performed in the cell-attached configuration. Borosilicate glass pipettes with a resistance of 2–3 MΩ were backfilled with internal solution, consisting of 125 mM NaCl, 3 mM KCl, 1.25 mM NaH_2_PO_4_, 0.85 mM CaCl_2_, and 20 mM HEPES, 100 nM (*S*)-glutamate, and 20 µM glycine, adjusted to pH 7.4. During the recordings, the cells were continuously flushed with external solution, consisting of 141 mM potassium gluconate, 2.5 mM NaCl, 11 mM EGTA, and 10 mM HEPES, adjusted to pH 7.4 by KOH. The antagonist solution was prepared by adding 1 µM (*R*)-OF-NB1. All solutions were adjusted to a DMSO concentration of 0.1%. NMDA currents were measured by clamping GluN1-1a/GluN2B or GluN1-1b/GluN2B wild-type expressing CHO cells at − 70 mV. The obtained data were filtered at 1 kHz using an 8-pole Bessel low-pass filter and an electrical interference filter at 50 Hz. Data analysis was performed and evaluated using Clampfit 10.7 (Molecular Devices, USA).

### Ligand Binding Assay

One day prior to transfection, human embryonic kidney (HEK) cells were reseeded into T75 flasks, aiming for a confluency of 70% after 24 h. HEK cells were then transiently co-transfected with the cDNAs (GluN1-1a/ eGFP, GluN1-1b/eGFP, GluN2B/pDsRed2-N1, GluN2B F176A/pDsRed2-N1, and GluN2B F194A/pDsRed2-N1) encoding the NMDAR subunits using Lipofectamine 3000 (Thermo Fisher Scientific, Germany) according to the manufacturer’s instructions. Approximately 60 h after transfection, the cells were resuspended in Dulbecco’s phosphate-buffered saline (PBS), and the positively double transfected cells were sorted using fluorescence-activated cell sorting (FACS). The membrane preparation, the protein yield determination, and ligand binding assay were performed as previously described [33]. Briefly, the affinity for NMDAR was evaluated using a receptor binding assay, in which racemic [^3^H], unlike ifenprodil (60 Ci/mmol; BIOTREND, Cologne, Germany), served as radioligand. The membrane fragments of NMDAR expressing HEK cells were incubated with 5 nM [^3^H]ifenprodil and TRIS/EDTA-buffer (5 mM TRIS/1 mM EDTA, pH 7.5) at 37 °C. To evaluate the unspecific binding, 10 µM unlabeled ifenprodil was used [33].

### Molecular Dynamics Simulations

All molecular dynamics (MD) simulations were performed using YASARA 20 via implemented macros, and the previously described cryo-EM (PDB ID: 6CNA) was used as starting structure for further modeling [22]. The implemented AMBER14 force field was used for simulation, with simulation durations ranging from 100 to 500 ns [34–36]. Simulation time steps were set to 1.35 fsec. The simulation box was “Cube”-shaped, extended the model min 10 Å to each side (extension = 10), and was filled with 0.9% NaCl at pH 7.4, using the TIP3P water model. The following settings were used: temperature at “298 K”, pressure at 1, density = 0.997, cutoff 8 Å-periodic cell boundary, and long-range coulomb forces (particle-mesh Ewald). The solute was prevented from diffusing around and crossing the periodic boundaries by CorrectDrift function [34, 35]. In order to speed up the simulation, the transmembrane domain (TMD) was removed according to Regan et al. [22]. All MD simulations were performed using the standard macro from YASARA “md_run.mcr”. Since exon 5 was only partially resolved in the Cryo-EM 6CNA, the missing 14 amino acids (^191^SKKRNYENLDQLSY^211^) were inserted via YASARA and moved to the start position for further simulations via MD simulation. For this purpose, the entire sequence of exon 5 was previously simulated in water, and then, the missing amino acids were attached to the ends of the resolved part of exon 5, Y204, and K190.

### Root Mean Square Fluctuation and Dynamic Cross Correlation Matrix

The root mean square fluctuation (RMSF) is the fluctuation of each molecule in relation to a reference point. In order to understand and compare the flexibility of the structures, the MD simulations were analyzed using the standard YASARA macro “md_analyze.mcr”. Additionally, the cross correlations of the crucial amino acids forming the ifenprodil binding pocket were calculated. The dynamic cross correlation matrices were calculated by using the same YASARA macro, “md_analyze.mcr”. The cross correlation indicates how the movements of all pairs are correlated; it can also indicate if two compared residues are in proximity. The values range from − 1 (completely anti-correlated) to 1 (completely correlated), a value of 0 indicates no correlation at all (if residues, e.g., in a binding pocket are in proximity, they should be correlated). A threshold of min. 0.9 was set for correlated residues, and the wild-type binding pocket correlation was set to 100%. To obtain the residual cross correlation in the mutants, the overall binding pocket correlation was calculated for the mutants with respect to the number of correlated residues in the wild-type binding pocket, respectively.

### Data Availability

Source data files are provided for Figs. 1, 2, 3, 4, 5, 6, and 8; Supplementary Figs. S1–S9; and Tables S1–5.

## Results

### (*R*)-OF-NB1 as a Promising GluN2B-Selective 3-Benzazepine Derivative

One aim of this study was to identify a compound that distinguishes between the two splice variants GluN1-1a and GluN1-1b to overcome off-target effects of unselective NMDAR antagonists. Further, the molecular mechanism underlying this splice variant selectivity was identified. *(R)-*OF-NB1 offered a high split between the two splice variants GluN1-1a and GluN1-1b (Fig. 1B–D). The IC_50_ value of *(R)-*OF-NB1 for the splice variant containing exon 5 is sevenfold higher than the IC_50_ value of GluN1-1a, 675 nM vs. 97 nM in *X. laevis* oocytes (Table S1). The hill slope and maximal inhibition highly differed between the splice variant isoforms. To understand which mechanisms result in these differences, the respective NMDAR’s binding pocket was investigated in detail on a molecular, electrophysiological, and mechanical level.

### Mutant Screening of Crucial Residues in the Ifenprodil Binding Pocket

**Fig. 2:**
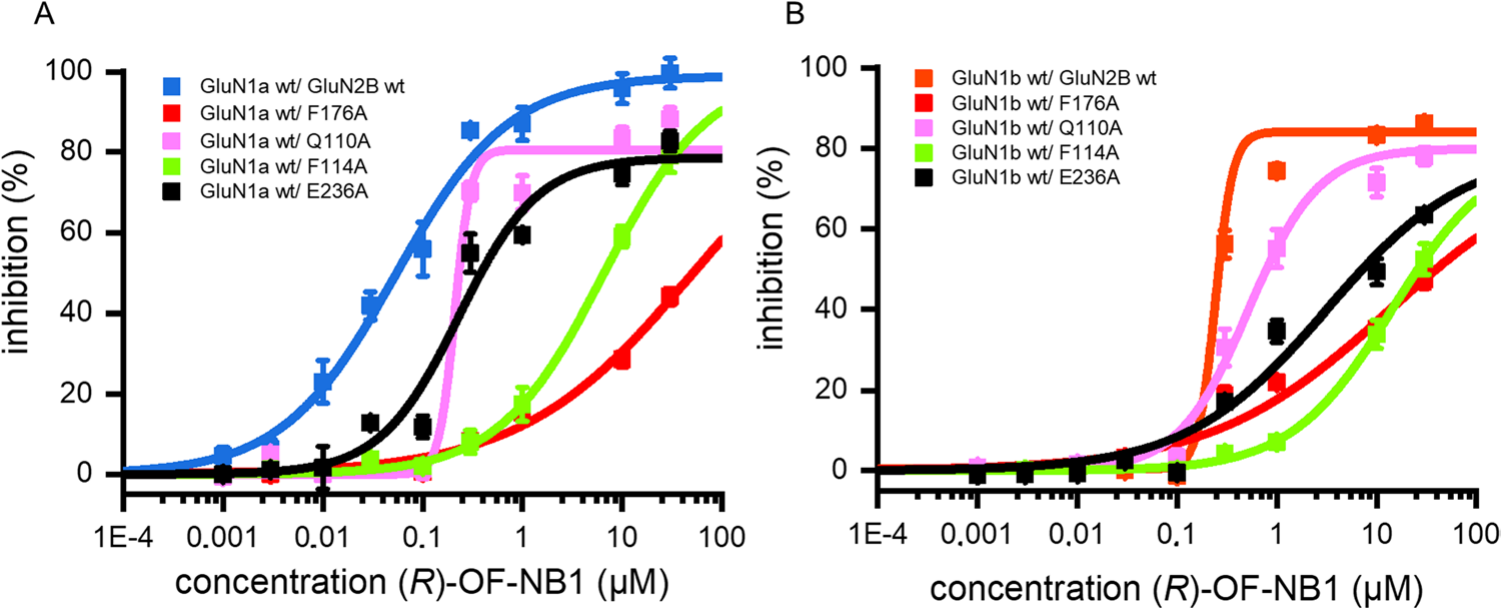
GluN2B wild type (GluN1a/GluN2B wt blue, GluN1b/GluN2B wt orange) vs. GluN2B F176A (red), Q110A (pink), F114A (green), and E236A (black). Dose response curves of (*R*)-OF-NB1 binding to GluN1b/GluN2B (**A**) and GluN1a/ GluN2B (**B**). Ion currents were activated by 10 µM glycine and 10 µM (S)-glutamate at GluN1/GluN2B expressing oocytes and inhibited by ascending (*R*)-OF-NB1 concentrations of 1, 3, 10, 30, 100, 300, 1000, 10,000, and 30,000 nM and recorded by TEVC. The inhibition (%) is plotted vs. (*R*)-OF-NB1 concentration (µM). Curves were generated from recordings of *n* = 4–10 independent oocytes for each wt or mutant. Higher concentrations of (*R*)-OF-NB1 were not tested due to its solubility limit of 30 µM. Hill slopes and A2 values are displayed in Table S2

Ifenprodil and its derivatives prevent the movement of the phenyl moiety 176 and thus lock the α5-helix to inhibit the receptor by interaction with F176 [28]. The substitution of F176 by alanine caused loss of activity and removed the split between the two splice variants (Fig. 2). Splice variant selectivity and function were least affected by mutations of Q110A, followed by E236A and F114A, which led to reduced activity with preserved splice variant selectivity (Fig. 2). For the receptor’s activity and splice variant selectivity, H-bond interactions formed by Q110 and E236 were less important than π-π interactions, especially those formed with F176.

The term allosteric interaction refers to reversible conformational changes of a specific protein site caused by modulation of another protein site. Those processes are relevant for cellular signaling and intercellular communication and are prominent in, i.e., NMDARs and other ion channels [37]. α5-helix movement is connected to NMDAR opening, which is impeded by flipping of F176. Further, a second phenylalanine is positioned at the lower α5-helix. To elucidate the relevance of the key residues for the receptor’s function, NMDARs consisting of GluN1-1a wt/GluN2B wt or GluN1-1b wt/GluN2B wt and NMDARs carrying the different mutations of interest were simulated and compared using YASARA structure. Here, the dynamic cross correlation matrices (DCCM) were evaluated, which display the correlation of movements of selected pairs corresponding to the residues in the simulated protein. To gain insight in differences in the binding pocket residues, the DCCM was investigated. A close proximity between two residues increases the likelihood of correlated dynamics between them. The dynamic cross correlation can range between − 1 (fully anti-correlated), 0 (no correlation), and + 1 (fully correlated). If residues are in close proximity and could potentially interact, for instance in a binding pocket, they display a dynamic cross correlation around 1.

**Fig. 3:**
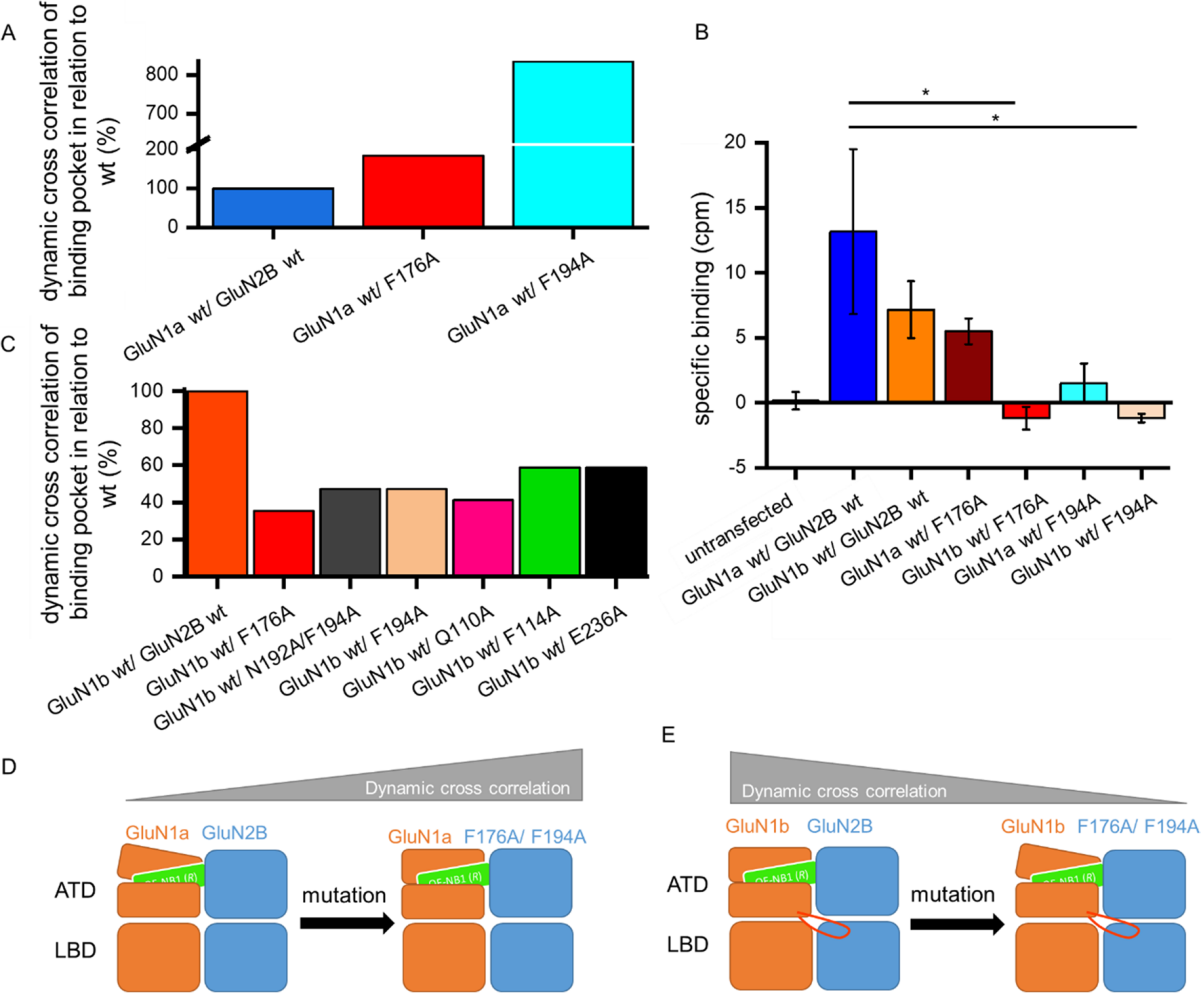
Cross correlation of the ifenprodil binding pocket increases in silico in GluN1a (**A**, **D**) or decreases in GluN1b (**C**, **E**). In vitro, the specific binding of ifenprodil decreases (**B**) after substitution with alanine. MD simulations of GluN1a wt/GluN2B wt (blue) vs. GluN1a/GluN2B F176A (red) and F194A (turquoise) bound to (*R*)-OF-NB1 (green square) revealed an increase of dynamic cross correlation between the amino acid residues forming the binding pocket, when the phenyl moiety is replaced by alanine (**A**,** D**). When the phenyl moiety is replaced by alanine in MD simulations, GluN1b wt/GluN2B wt (orange) vs. GluN1b/GluN2B F176A (red), Q110A (magenta), F114A (green), E236A (black), N192A/F194A (gray) and F194A (light pink) bound to (*R*)-OF-NB1 reveal a loss of dynamic cross correlation between the amino acid residues forming the binding pocket, followed by F114A and E236A, exon 5 is indicated by the orange loop (**C**, **E**). Ligand binding assays revealed that the specific binding of radiolabeled ifenprodil to NMDARs consisting of GluN1a wt/GluN2B wt is significantly higher compared to GluN1b/GluN2B F176A and F194A. NMDARs consisting of GluN1a wt/GluN2B wt display a higher specific binding than GluN1b wt/GluN2B wt. The specific binding was calculated by subtracting the non-specific binding in counts per minute (cpm) from the total binding events per minute. (*R*)-OF-NB1 is light green colored and exon 5 orange. Simulation durations were 100 ns (mutants) and 500 ns (wt)

Furthermore, in silico MD simulations revealed that a mutation of the phenyl moieties F176 and F194 to alanine decreases the cross correlation in the binding pocket drastically in NMDARs consisting of GluN1-1b/GluN2B (Fig. 3). However, comparing the cross correlation of the binding pocket for GluN1-1a/GluN2B wild type with the two phenyl moieties mutated to an alanine the cross correlation of the ifenprodil binding pocket is increasing compared to wild type (Fig. 3). Substitution of phenylalanines to alanine resulted in disruption of π-π interactions. In NMDAR lacking exon 5, the ifenprodil binding pocket-forming residues are more tightly coupled without binding of *(R)-*OF-NB1. The key residues move closer to each other, and the binding pocket itself is less voluminous. Upon *(R)-*OF-NB1 binding in silico, the pocket adopts a characteristic compound-bound configuration. Unlike GluN1-1a/GluN2B, GluN1-1b/GluN2B shows a coupling effect when *(R)-*OF-NB1 is binding in silico. The ifenprodil binding pocket-forming residues move closer upon binding of the compound.

Ligand binding assays revealed two distinct likelihoods of ifenprodil binding pocket interactions of NMDARs with and without exon 5. In line with the *(R)-*OF-NB1 dose response curves, in which GluN1-1b wt/GluN2B exerts lower sensitivity to 3-benzazepine inhibition, compared to GluN1-1a wt/GluN2B wt (Fig. 1B), binding assays displayed increased binding of radiolabeled ifenprodil to GluN1-1a wt/GluN2B compared to GluN1-1b/GluN2B (Fig. 3B). Further, in the in silico experiments, *(R)-*OF-NB1 exerts decoupling when binding to GluN1-1a wt/GluN2B wt and coupling when binding to GluN1-1b wt/GluN2B wt. Therefore, the GluN1-1a wt/GluN2B wt ifenprodil binding pocket is more accessible for the compound. In line with this and the electrophysiological recordings, GluN1-1a wt/GluN2B wt displayed higher specific ifenprodil binding compared to GluN1-1b wt/GluN2B (Fig. 3). The specific ifenprodil binding decreases in mutation F176A and F194A compared to GluN1-1a wt/GluN2B wt (Fig. 3B). This finding is consistent with results of electrophysiological mutant screening of crucial residues in the ifenprodil binding pocket, in which the F176A and F194A displayed a loss in inhibition by the 3-benzazepine derivative, *(R)-*OF-NB1 (Figs. 2 and 8, Table S1-2). Moreover, specific ifenprodil binding is reduced by alanine substitution of the phenylalanine moieties in each case, by F194A more extensive than by F176A. Furthermore, the phenylalanine GluN2B mutants in GluN1-1a result in a lower specific ifenprodil binding. In GluN1-1b, the phenylalanine mutations result in a greater loss of specific ifenprodil binding compared to GluN1-1a. These findings are also in line with the results of decreasing (GluN1-1b wt/GluN2B wt vs. F176A and F194A, Fig. 3C) or increasing (GluN1-1a wt/GluN2B wt vs. F176A and F194A, Fig. 3A) dynamic cross correlation (Fig. 3).

### Influence of Exon 5 on NMDAR Dynamics and Opening

According to Tian et al. [38], NMDAR antagonists like Zn^2+^ bind to the GluN2B ATD, which results in receptor closure because the ATD shifts into a relaxed configuration, with separation of GluN1 and GluN2B lobes, rolling down of the LBD inter-dimer interface and reduced tension of the TMD to promote receptor closing. Here, we simulate this state upon *(R)-*OF-NB1 binding.

**Fig. 4:**
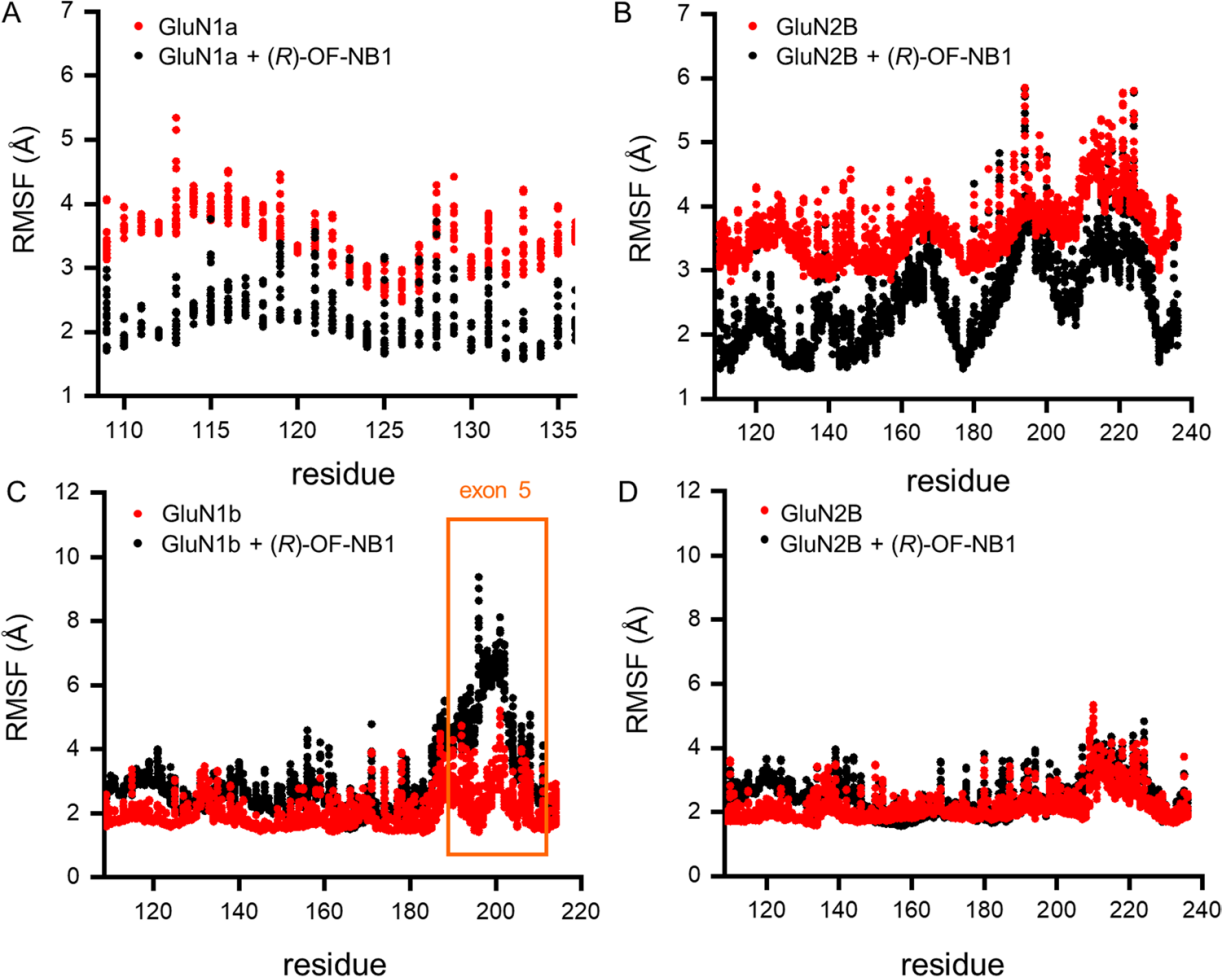
Root mean square fluctuations of GluN1a wt/GluN2B wt (**A**, **B**) and GluN1b wt/GluN2B wt (**C**, **D**) without compound (red dots) and *(R)-*OF-NB1 bound (black dots), and the position of exon 5 is marked by an orange box. MD simulations were performed using an AMBER14 force field, and simulation duration was 500 ns. The RMSF of the NMDAR without exon 5 showed a constant decrease when *(R)-*OF-NB1 is bound, while the RMSF of exon 5 increased when *(R)-*OF-NB1 is bound

**Fig. 5:**
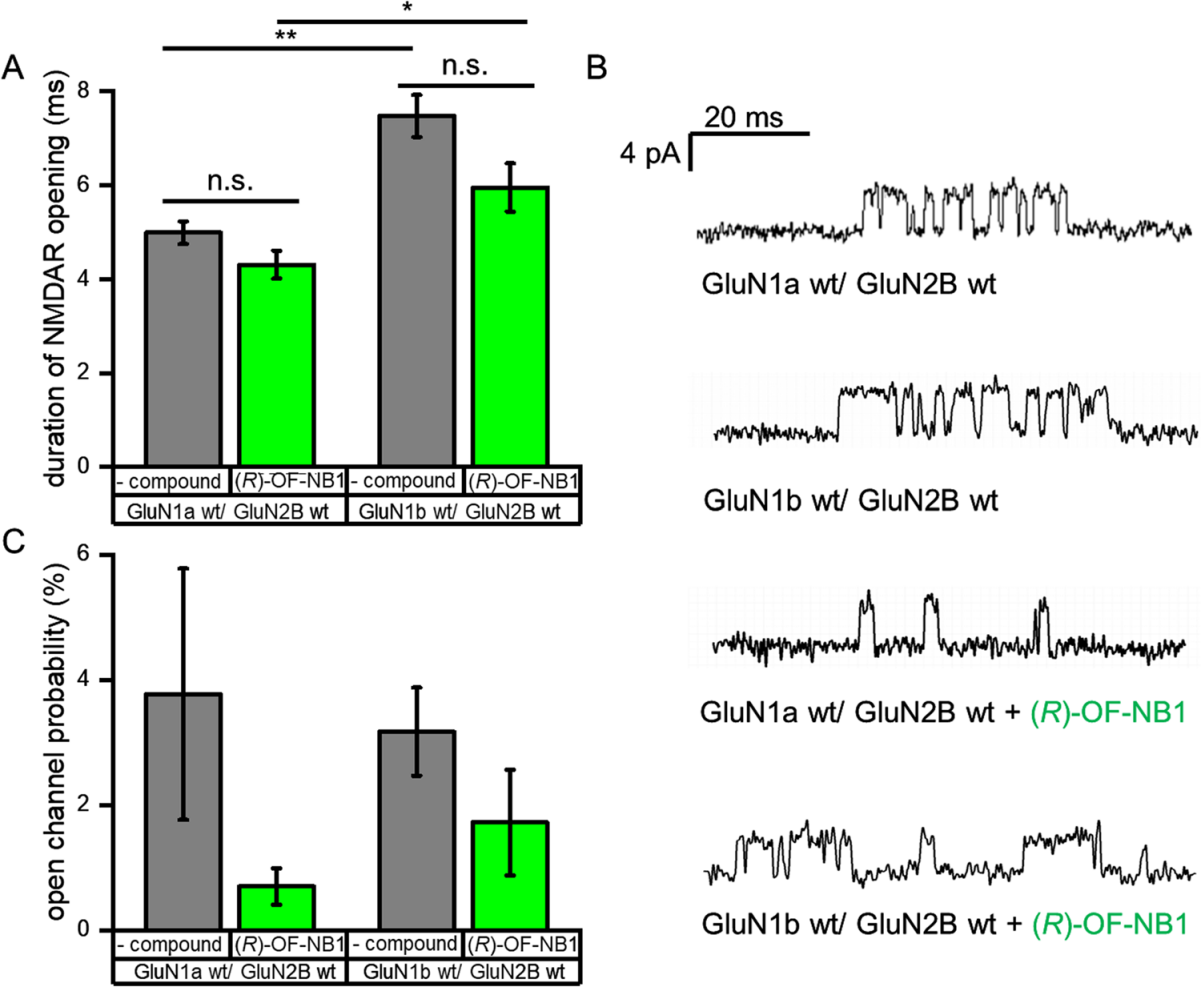
The duration of NMDAR opening is significantly increased by exon 5, compared to NMDAR without exon 5 present. The application *(R)-*OF-NB1 did not result in a significant reduction of the open time of NMDAR, neither when exon 5 was present nor without exon 5 (**A**). Exemplary single-channel traces of GluN1a/GluN2B and GluN1b/GluN2B expressing CHO cells, with and without application of 1 µM *(R)-*OF-NB1, recorded in cell attached configuration with a single-channel conductance of around 50 pS (**B**). Open channel probabilities indicate that exon 5 may shift the open probability of the NMDAR; towards open channel conformations, these effects did not reach the level of significance (**C**). Traces were generated from recordings of *n* = 3–5 independent GluN1/GluN2B expressing CHO cells

The root mean square fluctuation (RMSF) of the NMDAR without exon 5 (GluN1-1a) is reduced upon *(R)-*OF-NB1 binding. The binding of the compound forces the receptor into closed state conformation that is more stable (Fig. 4). The structure itself becomes more flexible, preventing the rolling motion of two LBD dimers, and there is an increase of the RMSF of exon 5 when the NMDAR is bound to *(R)-*OF-NB1. Exon 5 shifts to increase flexibility upon binding of the compound interfering with the relaxed conformation of the ATD, the separation of the GluN1 and GluN2B lower lobes, and the reduction of the tension of the TMD. Single-channel patch clamp recordings showed that exon 5 increases the mean open time of NMDAR (Fig. 5A and B). Exon 5 presumably shifts the open probability of the NMDAR towards open-channel conformations (Fig. 5C).

### Exon 5 Is a Positive Allosteric Modulator of NMDARs Acting Similar as Spermine

**Fig. 6:**
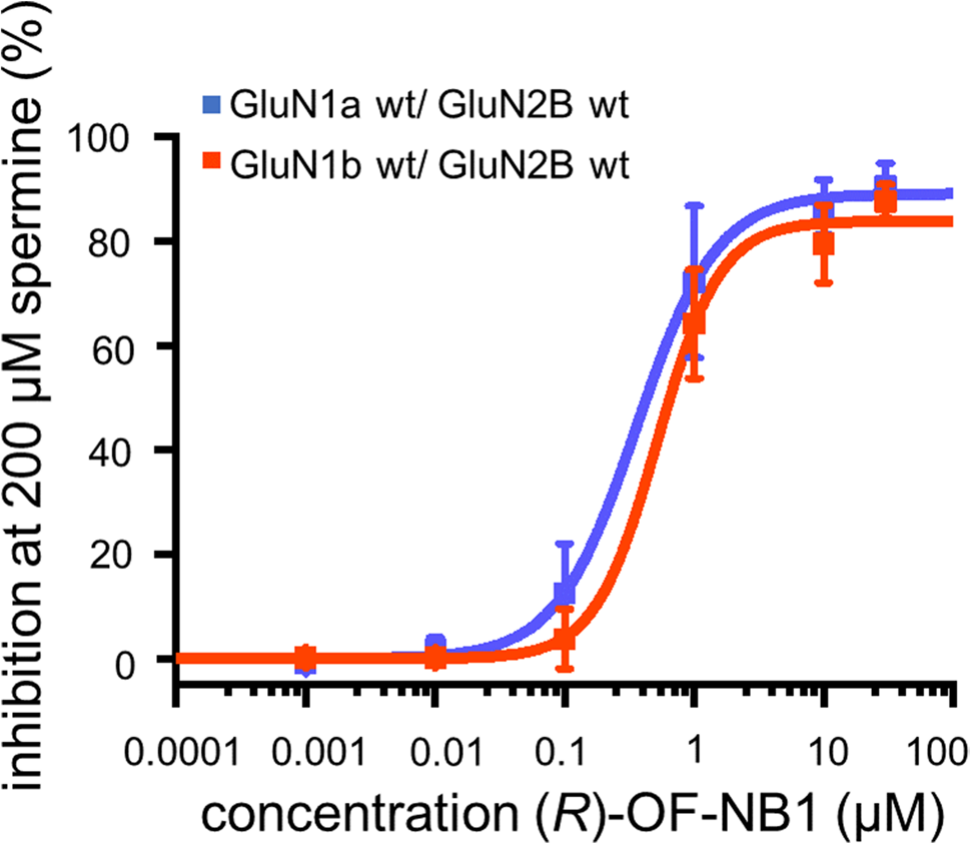
Dose response curves of mean inhibitions (± SEM) of GluN1a/GluN2B (blue) vs. GluN1b/ GluN2B (orange) at ascending *(R)-*OF-NB1 concentrations in the presence of 10 µM glycine and 10 µM (*S*)-glutamate and 200 µM spermine. The inhibition in the presence of agonists and spermine (%) was plotted against the *(R)-*OF-NB1 concentration (µM). Spermine abolished the splice variant selectivity of *(R)-*OF-NB1 by decreasing its inhibitory effect against Glu1a/ GluN2B. Hill slopes and A2 values are displayed in Table S3

The closure of the NMDAR is mediated by tension introduced in the TMD from a rolling motion of the LBD caused by conformational changes in the ATD upon compound binding, as demonstrated by Esmenjaud et al. [39]. It was found that spermine as an allosteric modulator prevented this rolling motion. The NMDAR is probably modulated by polyamines such as spermine and spermidine in a similar way to exon 5 [22]. As described above, we recorded GluN1-1a/GluN2B and GluN1-1b/GluN2B expressing oocytes with 200 µM spermine and ascending concentrations of *(R)-*OF-NB1, to examine if the allosteric effects are mechanically in conjunction or can be potentiated. Using spermine, we were able to abolish the split between the splice variants (Fig. 6, Table S1-3). By using spermine, *(R)-*OF-NB1 was shifted to have the same inhibitory effect on GluN1-1a/GluN2B expressing oocytes as exon 5 does naturally (Fig. 6). The IC_50_ of GluN1-1b/GluN2B expressing oocytes in the presence of *(R)-*OF-NB1 and agonists with and without spermine were similar, ruling out further potentiation of the allosteric effect of exon 5.

### Trapping the α5-Helix at the GluN1/GluN2B Interface Results in Deactivation of the NMDAR

**Fig. 7:**
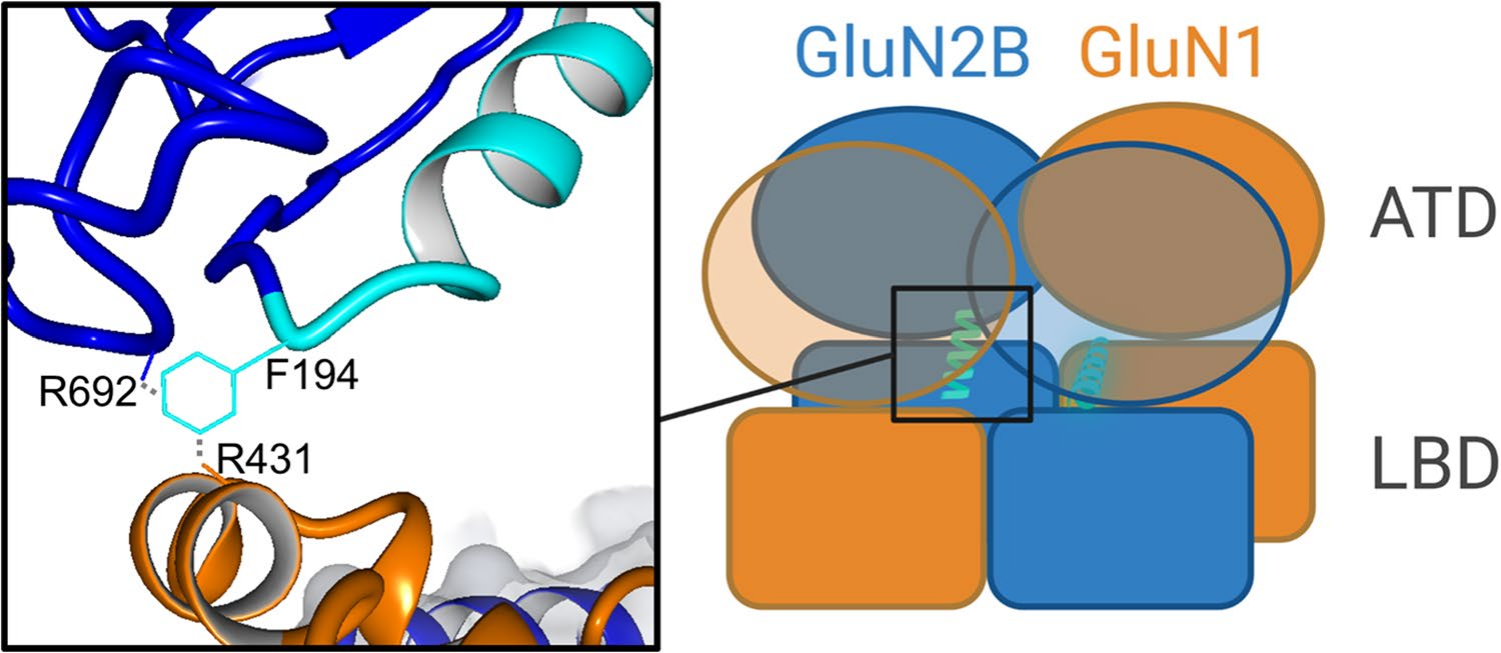
GluN1/GluN2B at the ATD and LBD interface. GluN1 is orange, and GluN2B is blue colored. The key amino acid interaction between the GluN2B ATD and LBD layers at the end of α5-helix (light blue) was also found to structurally connect the ATD and LBD interface of the GluN1 and GluN2B subunit. F194 shows cation-π interactions with R692 and R424. 6CNA served as template structure, and structure was edited in YASARA. Scheme was rendered with BioRender

According to Lü et al. [29], interactions between amino acid residues N192 and F194 at the C-terminal end of α5-helix were also found to link ATD and LBD interface of GluN1 and GluN2B subunit structurally. F194 shows cation-π interactions with R725 (R692) and R431 (R424) in silico (Fig. 7). To understand the allosteric downstream pathway of the NMDAR by *(R)-*OF-NB1 and its splice variant selectivity, differences in their cross correlation and RMSF, the key residues GluN2B N192, F194, and N192 plus F194 were substituted with alanine as single or double mutant and tested in TEVC experiments.

**Fig. 8:**
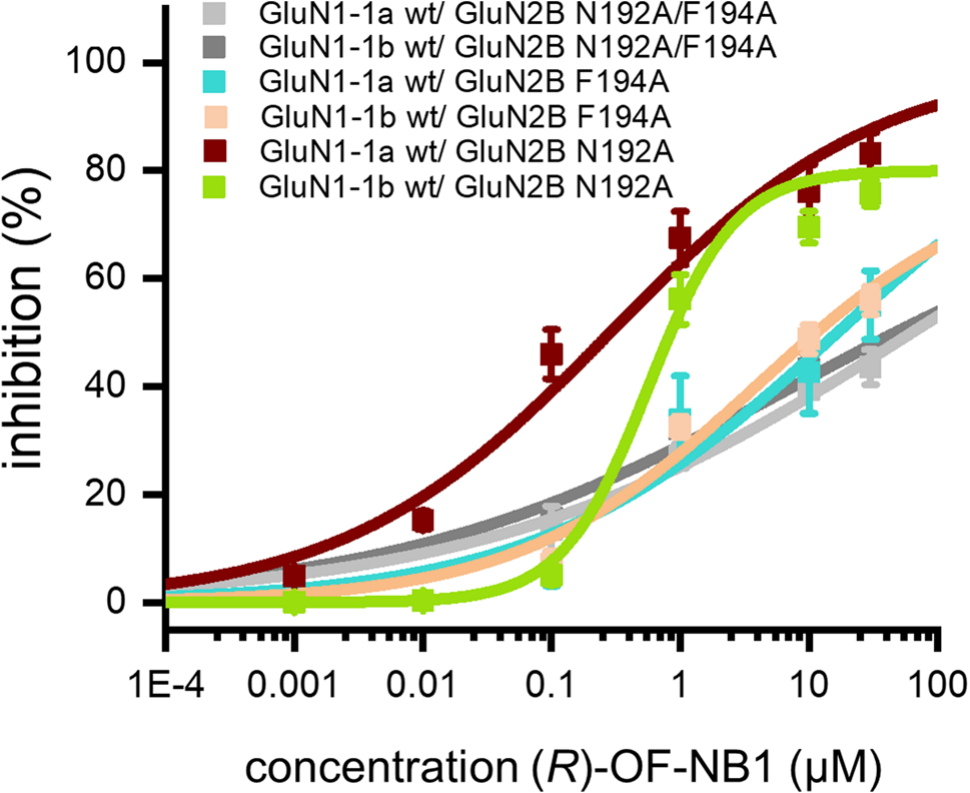
Mean inhibition ± SEM of ion current in TEVC recordings at GluN1 wt/GluN2B N192A/F194A (gray), GluN1 wt/ GluN2B N192A (dark red and light green), and GluN1 wt/ GluN2B F194A (turquoise and light pink) expressing oocytes by ascending *(R)-*OF-NB1 concentrations in presence of 10 µM glycine and 10 µM (*S*)-glutamate. The inhibitions (%) are plotted against the respective concentration (µM). Data were fitted to a Hill equation and the fits are shown. Curves were generated from recordings of *n* = 6–9 independent oocytes for each mutant. Mutation of GluN2B wt to GluN2B F194A revealed a drastic loss in splice variant selectivity and inhibition. 30 µM is the limit of *(R)-*OF-NB1 solubility and higher concentrations could not be tested. Hill slopes and A2 values are displayed in Table S2

In TEVC recordings, GluN2B N192A/F194A or F194A were coexpressed with GluN1-1a or GluN1-1b. The mutants GluN2B N192A/F194A and GluN2B F194A reduced inhibition and led to a complete loss of split between the two splice variants (Fig. 8). The phenotype of the determined inhibition is wt-like after mutagenesis of GluN2B N192A (Fig. 8). Our results rule out the possibility that downstream allosteric modulation of the NMDAR is associated with the predicted hydrophobic interaction between the asparagine at the lower α5-helix and the LBD [29]. Interestingly, the observed phenotype of the F176A located in the ifenprodil binding pocket was very similar to the loss of activity and splice variant selectivity of the F176A (Fig. 2). In silico, GluN2B F194A reduced dynamic cross correlation as drastically as GluN2B F176A (Fig. 3). Considering the same effect was shown by the double mutant (N192A/F194A), the phenylalanine appears to be the crucial interaction point between the ATD and LBD interfaces. As a result of 3-benzazepine binding to the ifenprodil binding pocket at the ATD-LBD interface resulting in receptor closure via TMD, F194 is likely to influence this allosteric pathway.

## Discussion

We discovered the 3-benzazepine *(R)-*OF-NB1 as a promising GluN2B-selective ifenprodil derivative. The activity of highly potent compounds depends on π-π interactions with the aromatic amino acids GluN2B F176, according to the existing pharmacophore model [40, 41]. Additionally, *(R)-*OF-NB1 clearly discriminates the two splice variants, GluN1-1a and GluN1-1b. Despite inhibition of GluN1-1a/GluN2B expressing oocytes by low *(R)-*OF-NB1 concentrations, GluN1-1b/GluN2B expressing oocytes still showed activity. These exon-5-carrying NMDARs have been shown to be less sensitive to ifenprodil and spermine [12]. However, at higher *(R)-*OF-NB1 concentrations, GluN1-1b/GluN2B expressing oocytes exerted sensitivity (Fig. 1).

### Impact of Exon 5 on the NMDAR Activity

**Fig. 9:**
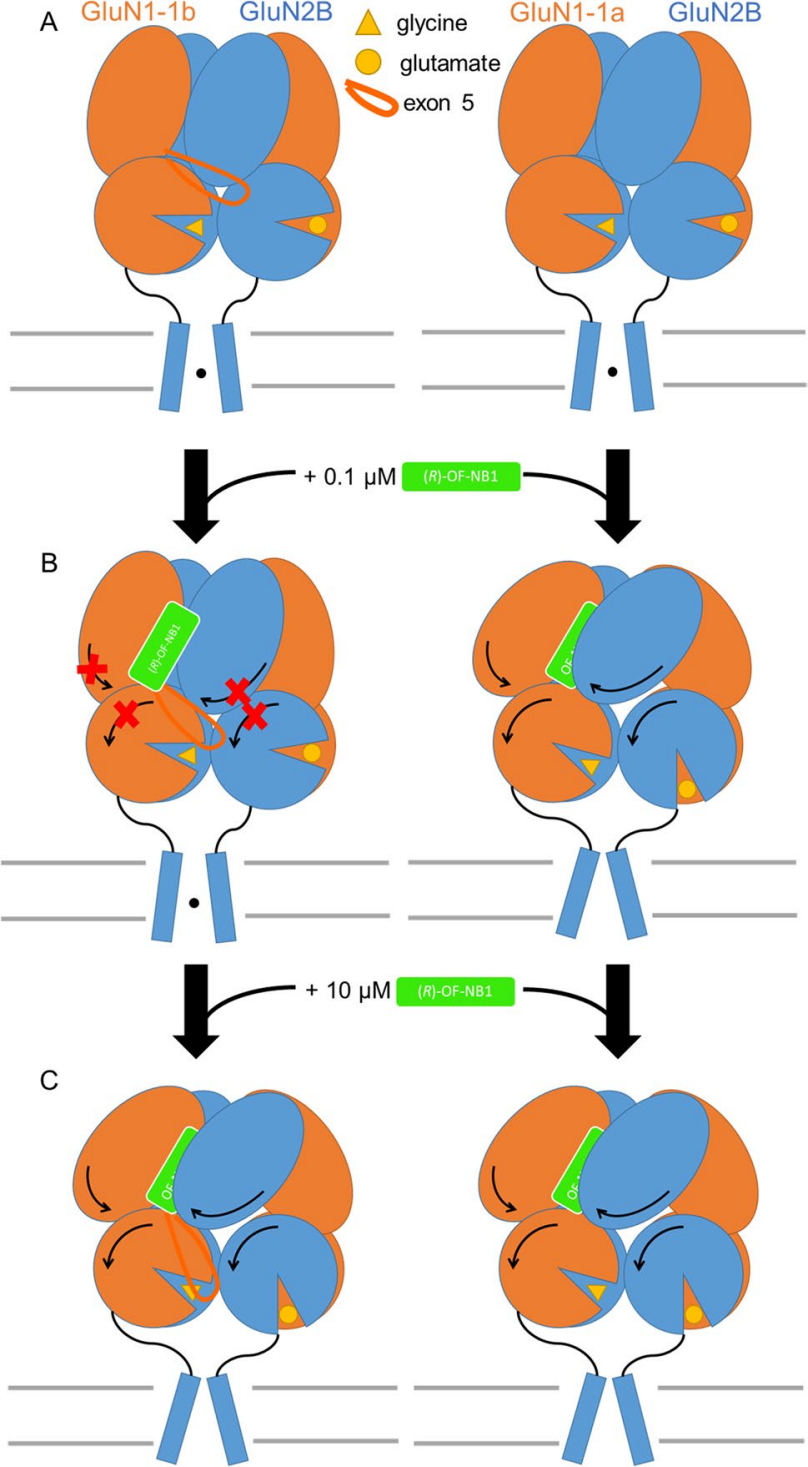
Two distinct allosteric modulations occur in NMDARs, consisting of GluN1a/GluN2B or GluN1b/GluN2B. GluN1 is orange, and GluN2B is blue colored. In NMDAR without exon 5 (right), the ifenprodil binding pocket is accessible to the antagonist *(R)-*OF-NB1 (green). The binding pocket is deflated, and the compound can easily slip into its binding site and inhibit the NMDAR. In NMDAR with exon 5 (left), amino acid residues are more separated to prevent ifenprodil binding pocket formation and prevent inhibition. Both NMDAR splice variants are activated by their respective agonists, glycine and (*S*)-glutamate (yellow) (**A**). NMDARs without exon 5 are already inhibited at lower concentrations of the antagonist *(R)-*OF-NB1. Since exon 5 becomes highly flexible when the NMDAR is bound to *(R)-*OF-NB1, NMDARs carrying exon 5 are not inhibited at low concentrations of this antagonist (**B**). At higher *(R)-*OF-NB1 concentration, the movement of exon 5 is not sufficient anymore to prevent the inhibition, and both NMDAR splice variants are inhibited (**C**)

MD simulations revealed that *(R)-*OF-NB1 has a decoupling effect of the ifenprodil binding pocket-forming amino acids in NMDAR, consisting of GluN1-1a/GluN2B. However, in NMDAR carrying exon 5 (GluN1-1b), binding of *(R)-*OF-NB1 showed the exact opposite effect, a coupling of the amino acids forming the ifenprodil binding pocket. Already before binding of an antagonist to the ifenprodil binding site, the binding pockets exhibit different properties; in NMDARs lacking exon 5, the ifenprodil binding pocket-forming residues are closer together, and the binding pocket is deflated (Fig. S1). When *(R)-*OF-NB1 is bound, the pocket expands into a compound-bound configuration. In NMDARs with exon 5, the residues forming the ifenprodil binding pocket are less correlated without the compound and more strongly coupled upon the binding of the compound (Fig. 9).

Tian et al. [38] suggested both GluN1-GluN2 and GluN2-GluN2 subunit interactions as a major impetus for allosteric transduction in NMDARs. GluN1/GluN2B ATD are prone to conformational changes and exhibit unique interdomain coupling mechanisms regulated crucially by the LBD interdimer interface conformation [30, 42]. Activation and inhibition of NMDARs composed of GluN2B are regulated by LBD rolling, which is promoted by ATD conformational changes. These rolling motions change the tension on the TMDs and lead to receptor opening or closing. A rolling down of the LBD decreases the tension of the LBD-TMD linkers and eventually results in the closing of the NMDAR [39, 43]. The ATD-LBD interface is an important target for NMDAR regulation, including the sensitivity of protons and the deactivation rate of NMDARs. The exon 5 motif acts as a naturally embedded ligand in the receptor’s sequence to control the GluN1-GluN2B LBD subunit interface, which in turn regulates the TMD [22, 42]. Compounds like spermidine or structures like exon 5 are positive allosteric modulators of the NMDAR. They stabilize a compact conformation of the ATDs and therefore promote the open state of the NMDAR [38]. Since exon 5 and polyamines like spermine and spermidine show similar modulatory effects on NMDARs, it is likely that exon 5, as a tethered ligand, and these polyamines bind and modulate the identical domain interfaces. Both are able to result in reduced proton sensitivity and faster deactivation rates [24, 44]. A similar mechanism could also apply to several subtype-specific compounds, such as PYD106, that are likely to bind at ATD-LBD interface as well [45].

In accordance with Vance et al. [46], exon 5 most likely promotes the opening probability of the NMDAR (Fig. 5C). This suggests that the receptor is already shifted towards an open state pathway with crucial amino acid residues more separated, thereby preventing the formation of the ifenprodil binding pocket (Figs. 3 and 4). Consistent with this hypothesis, Tajima et al. [43] found the apo state of the NMDAR consisting of GluN1b and GluN2B being likely in the active conformation. In NMDARs lacking exon 5, the ifenprodil binding pocket conformation is favored, and the apparent affinity of *(R)-*OF-NB1 is increased.

NMDARs lacking exon 5 showed a continuous decrease in RMSF when bound to *(R)-*OF-NB1. The receptor is more flexible without the compound, and the compound forces the receptor into a stable closed conformation in silico (Fig. 4). The open time of the NMDAR with exon 5 is increased (Fig. 5A). Additionally, the sensitivity of GluN1-1b/GluN2B for open-channel active compounds is increased (Fig. 5A). Exon 5 likely shifts the NMDAR to open conformations and facilitates NMDAR opening (Fig. 5A, C). When the NMDAR is bound to *(R)-*OF-NB1, the structure itself becomes more flexible to reduce rolling motion between the two LBD dimers. There is an increase in the RMSF of exon 5 (residues K190–A212, Fig. 4). The flexibility of exon 5 increases upon compound binding and disrupts the relaxed configuration of the ATD, the separation of the lower lobes of GluN1/GluN2B, and the reduction in tension at the TMD. To summarize, exon 5 acts like a ligand to control the GluN1-GluN2B LBD interface, which in turn regulates the TMD, and thus the ion channel activity. Extracellular polyamines, including spermine, bind to GluN2B-containing NMDARs, reducing proton inhibition and thereby enhancing receptor activity [23, 47–49]. Spermine potentiates NMDARs carrying exon 5 less than NMDARs without exon 5, according to Yi et al. [50]. Nevertheless, we rule out spermine competing with exon 5; rather, it is not shifting the NMDAR further towards open conformations (Fig. 6). We conclude that the abolished splice variant selectivity and the shift in inhibitory effect of *(R)-*OF-NB1 on GluN1-1a/GluN2B expressing oocytes are due to the positive allosteric modulation of spermine. Here, spermine positively stimulated NMDARs composed of GluN1-1a/GluN2B to the same extend as exon 5, which acts as a naturally embedded positive allosteric modulator. NMDARs are positively modulated by exon 5 in the same manner as by spermine. Our findings are in line with those of Traynelis et al. [23] and Masuko et al. [51], who identified exon 5 as a constitutive spermine-like modulator that is slightly larger than the α5-helix and shields or directly interacts with the spermine binding site.

Ifenprodil locks the α5-helix in the closed conformation, resulting in inhibition by a “foot-in-the-door” mechanism [28]. We found that mutation of GluN2B wt to F176A resulted in loss of activity and splice variant selectivity (Fig. 2). Additionally, NMDARs containing GluN1-1b/GluN2B showed drastically reduced cross correlation in the binding pocket when the phenyl moieties F176 and F194 were mutated to alanine (Fig. 3). However, a comparison of the cross correlation of the ifenprodil binding pocket with the wild type and the two phenyl moieties mutated to alanine indicates that the cross correlation increases compared to the wild type (Fig. 3). The ligand binding assays support the hypothesis of two distinct 3-benzazepine binding dynamics of NMDARs consisting of GluN1-1a/GluN2B and GluN1-1b/GluN2B. GluN1-1a is more sensitive to 3-benzazepines, the binding pocket is more easily formed, and *(R)-*OF-NB1 decoupled the binding pocket; first, it is deflated, then the binding pocket is formed, and the pocket is uncoupled and no longer deflated (Fig. S1). GluN1-1b is uncoupled without *(R)-*OF-NB1 and is coupled by the compound, and the receptor is shifted from dynamic equilibrium towards open states. Accordingly, GluN1-1a shows more specific ifenprodil binding than GluN1-1b. F176A and F194A mutations in GluN1-1a lead to an increase in dynamic cross correlation, whereas they decrease the dynamic cross correlation in GluN1-1b. Specific ifenprodil binding is decreased by mutation of the phenylalanine moieties to alanine, respectively, by F194A more than by F176A. In GluN1-1a, the phenylalanine mutants result in lower specific ifenprodil binding and increase the dynamic cross correlation, thus having a coupling effect. The ifenprodil binding pocket is decoupled in wt by *(R)-*OF-NB1, and the receptor is inhibited. In GluN1-1b, compared with GluN1-1a, the phenylalanine mutations lead to a greater loss of specific ifenprodil binding and a decrease in dynamic cross correlation, i.e., a decoupling, so that the compound binding is reduced. Binding of *(R)-*OF-NB1 to exon 5 containing wt NMDARs has a coupling effect and is abolished by the mutants (Fig. 3). The mutants cause the opposite effect of the normal influence of *(R)-*OF-NB1 on the ifenprodil binding pocket and thus presumably can abolish the split and inhibition. These results strongly indicate that both phenyl moieties are crucial for NMDAR activity and 3-benzazepine binding.

### Downstream Allosteric Modulation of the α5-Helix

**Fig. 10:**
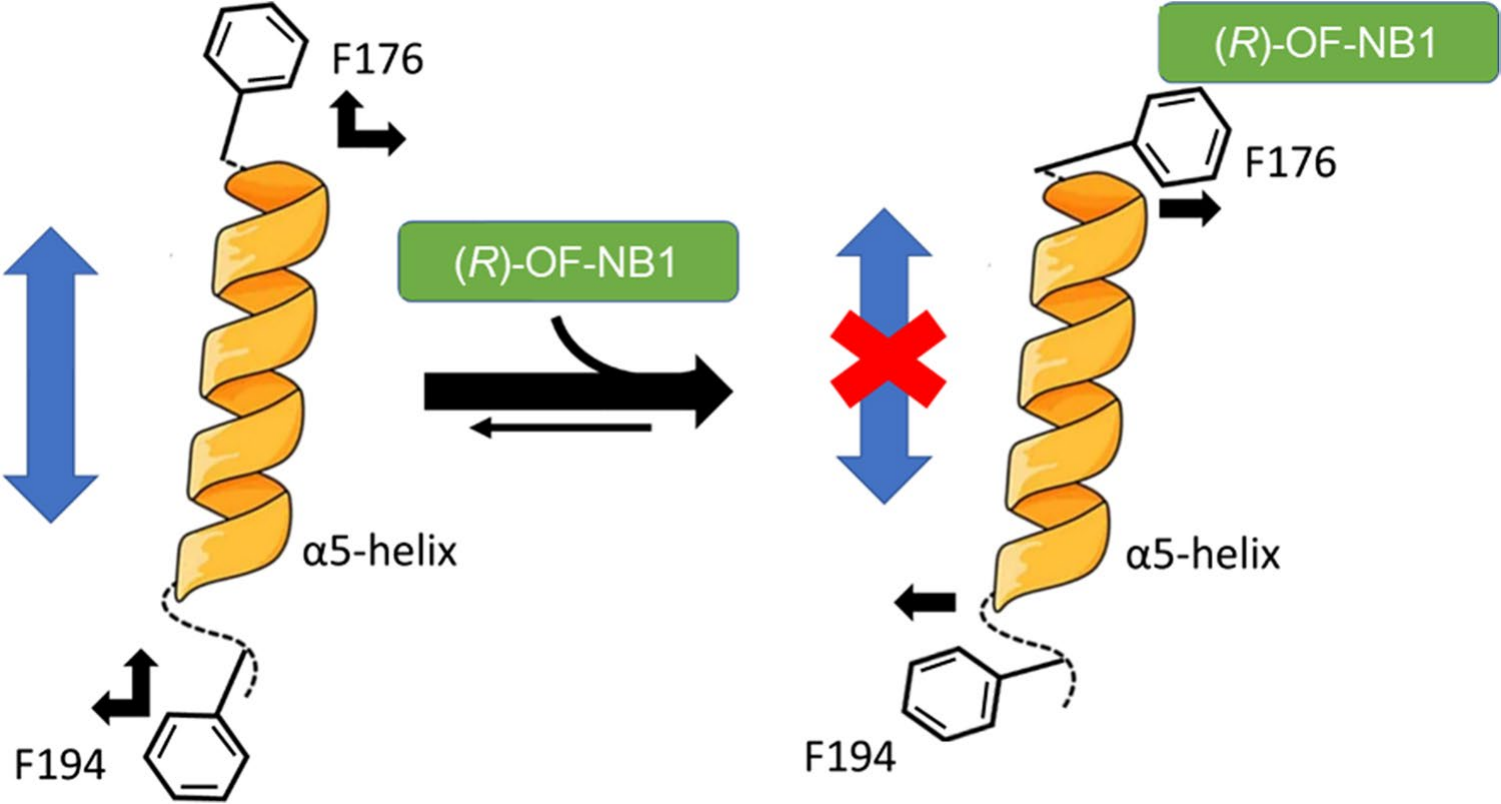
Proposed inhibitory mechanism at the ifenprodil binding site. Negative allosteric modulators like *(R)-*OF-NB1 bind to F176 to trap the α5-helix (orange) in close-inhibited conformation, which results in inhibition. F176 is in a vertical position in an open uninhibited state and allows movement [28]. When deactivated and/ or inhibited, the orientation of F176 is in a horizontal position. F194 is located downstream at the end of α5-helix and acts via an analogous mechanism when the compound is bound. Both phenyl moieties are able to lock the α5-helix and therefore trap it in its movement when the compound forces these moieties to flip to a horizontal position in silico

The α5-helix is important for transition from the unbound towards deactivated state with ifenprodil-like compounds bound. When the NMDAR is in an active state, F176 is orientated perpendicular to the receptor, and the GluN2B subunit is oriented towards the GluN1 subunit. Upon binding of an ifenprodil-like compound, the receptor transitions into the deactivated state and the GluN2B subunit repositions. A proposed ~ 90° flip of the phenyl moiety from a vertical (active state) to a horizontal state is associated with NMDAR deactivation [28]. Furthermore, the α5-helix is flanked by another phenyl moiety located downstream at the ATD-LBD interface (F194) [29]. Since the mutation of GluN2B F176 and GluN2B F194 to alanine exerts similar reduced inhibition and loss of splice variant selectivity in TEVC recordings (Figs. 2 and 8), we propose a common mechanism at the beginning and end of the α5-helix. When the NMDAR is in an active state, the F194 is orientated vertically to the receptor, and the GluN2B subunit is oriented towards the GluN1 subunit in silico. During binding of an ifenprodil-like compound, the receptor enters the deactivated state, and F194 flips horizontally, like F176, resulting in the α5-helix being trapped in a closed-inhibited state, preventing its movement (Fig. 10).

Here, we unraveled the allosteric splice variant-dependent mechanism of optimized 3-benzazepine inhibitors on NMDARs. Furthermore, we have uncovered the molecular mechanism that allows physiological and pharmacological modulation distinguishing between GluN1 splice variants carrying exon 5, thus defining ligand binding and dynamics of inhibition of NMDARs by 3-benzazepines in general. This highly complex mechanism accounts for the demonstrated specificity of (*R*)-OF-NB1 and 3-benzazepines in general, and we therefore exclude apparent cooperativity of (*R*)-OF-NB1. Further, exon 5 prevents a 100% inhibition by its flexibility, which could lead to the α5-helix not being fully trapped. Overall, we provide a useful tool to investigate the role of NMDARs in health and disease by enabling more highly targeted pharmacology.

### Role of (*R*)-OF-NB1 as Biomarker

Ahmed et al. [52] characterized ^18^F-OF-NB1 as a promising positron emission tomography (PET) probe for selectively targeting of GluN2B subunits. ^18^F-OF-NB1 was shown to be accumulated in GluN2B-rich brain regions, which was enabled by a high blood–brain barrier permeability of the compound. In ex vivo biodistribution assays, ^18^F-OF-NB1 displayed a high cerebellar accumulation [52–56]. According to Ahmed et al. [57], in vivo PET probe characterizations of (*R*)-^18^F-OF-NB1 in non-human primates revealed a high uptake, slow washout kinetics, and a high cingulate cortex accumulation. The *R*-enantiomer, (*R*)-^18^F-OF-NB1, exerted a four- to five-fold higher affinity in vivo than the *S*-enantiomer, (*S*)-^18^F-OF-NB1 [57]. Therefore, ^18^F-OF-NB1 is a promising GluN2B radioligand for use in PET imaging studies in patients with NMDAR-related neurodegenerative diseases.

### Supplementary Information

Below is the link to the electronic supplementary material.

Supplementary file1 (DOCX 283 KB)

Supplementary file2 (XLSX 794 KB)

## Data Availability

All data generated or analyzed during this study are included in this article, its supplementary information files and the source data.
