# Supplementary material for: Downstream Allosteric Modulation of NMDA Receptors by 3-Benzazepine Derivatives
Source: Mol Neurobiol. 2023 Aug 5;60(12):7238–52. doi: 10.1007/s12035-023-03526-1 (PMC10657792; doi:10.1007/s12035-023-03526-1)
Supplement: Supplementary file 1 — Supplementary file1 (DOCX 283 KB) [file 12035_2023_3526_MOESM1_ESM.docx]

Supplementary information


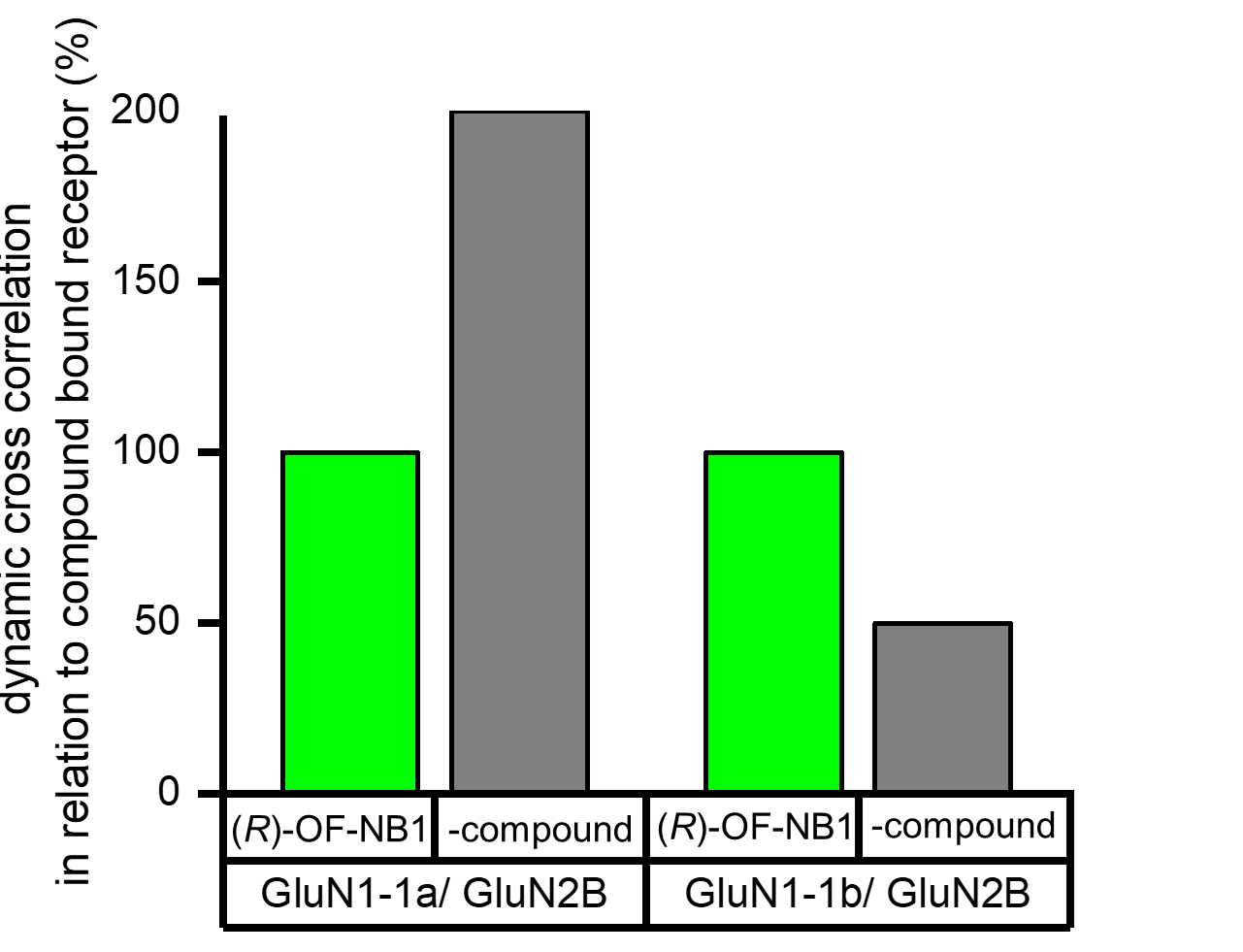


Figure S1: Dynamic cross correlation of the ifenprodil binding pocket increases upon binding of (*R*)-OF-NB1 *in silico* in GluN1b or decreases in GluN1a upon (*R*)-OF-NB1. In exon 5 carrying NMDARs the residues are more coupled without (*R*)-OF-NB1, while in NMDARs without exon 5 (*R*)-OF-NB1 works as dynamic decoupler in the binding site. Thus, the ifenprodil binding pocket deflates upon (*R*)-OF-NB1 binding in NMDARs without exon 5, while it inflates upon (*R*)-OF-NB1 binding in NMDARs with exon 5.

Table S1: Inhibitory effects of the different compounds on GluN1a or GluN1b subunit containing receptors. IC_50_ values, Hill coefficients and A_2_ values (level of maximal inhibition) derived from the fitted curves.

| subunit | IC_50_ (nM) | SD (nM) | Hill coefficient | SD | A2 (%) | SD (%) | n |
| --- | --- | --- | --- | --- | --- | --- | --- |
| GluN1a wt/ GluN2B wt | 97.0 | 72.8 | 0.9 | 0.3 | 100.5 | 12.1 | 10 |
| GluN1b wt/ GluN2B wt | 675.2 | 172.1 | 6.2 | 2.7 | 84.8 | 2.1 | 7 |

Table S2: Inhibitory effects of (*R*)-OF-NB1 on the GluN1a and GluN1b together with the GluN2B mutants, in the presence of the respective agonists. IC_50_ values, Hill coefficients and A2 values (level of maximal inhibition) derived from the fitted curves.

| subunit | IC_50_ (nM) | SD (nM) | Hill coefficient | SD | A2 (%) | SD (%) | n |
| --- | --- | --- | --- | --- | --- | --- | --- |
| GluN1a wt/ GluN2B F176A | 51264.3 | 108253.9 | 0.5 | 0.1 | 100 | 0 | 11 |
| GluN1b wt/ GluN2B F176A | 13588.9 | 1755.4 | 0.5 | 0.03 | 80 | 0 | 10 |
| GluN1a wt/ GluN2B F114A | 6575.4 | 2922.3 | 0.8 | 0.1 | 100 | 0 | 5 |
| GluN1b wt/ GluN2B F114A | 14199.6 | 1276.6 | 0.9 | 0.1 | 80 | 0 | 6 |
| GluN1a wt/ GluN2B E236A | 231.7 | 35.6 | 1.1 | 0.2 | 100 | 0 | 5 |
| GluN1b wt/ GluN2B E236A | 3145.1 | 430.2 | 0.6 | 0.1 | 80 | 0 | 5 |
| GluN1a wt/ GluN2B Q110A | 210.3 | 30.3 | 5.4 | 2.0 | 100 | 0 | 6 |
| GluN1b wt/ GluN2B Q110A | 518.7 | 47.5 | 1.2 | 0.1 | 80 | 0 | 5 |
| GluN1a wt/ GluN2B F194A | 16647.8 | 8203.1 | 0.4 | 0.3 | 100 | 0 | 4 |
| GluN1b wt/ GluN2B F194A | 3844.9 | 697.5 | 0.5 | 0.05 | 80 | 0 | 7 |
| GluN1a wt/ GluN2B N192A/F194A | 63117.4 | 33688.7 | 0.3 | 0.1 | 100 | 0 | 4 |
| GluN1b wt/ GluN2B N192A/F194A | 7516.9 | 2093.8 | 0.3 | 0.04 | 80 | 0 | 6 |
| GluN1a wt/ GluN2B N192A | 282.1 | 61.8 | 0.4 | 0.03 | 100 | 0 | 10 |
| GluN1b wt/ GluN2B N192A | 566.7 | 57.1 | 1.3 | 0.1 | 80 | 0 | 14 |

Table S3: Inhibitory effects of (*R*)-OF-NB1 on the GluN1a and GluN1b subunit in the presence of the respective agonists and 200 µM spermine. IC_50_ values, Hill coefficients and A2 values (level of maximal inhibition) derived from the fitted curves.

| subunit | IC_50_ (nM) | SD (nM) | Hill coefficient | SD | A2 (%) | SD (%) | n |
| --- | --- | --- | --- | --- | --- | --- | --- |
| GluN1a wt/ GluN2B wt | 359.4 | 33.0 | 1.4 | 0.1 | 89.1 | 1.5 | 15 |
| GluN1b wt/ GluN2B wt | 521.7 | 50.9 | 1.8 | 0.2 | 83.8 | 1.4 | 10 |

Table S4: P-values of mean open times of NMDARs, obtained by single channel recordings of NMDAR containing and without exon 5 (GluN1-1b/ 2B) before in the presence and absence of 1 µM (*R*)-OF-NB1. A p-value <0.050 is significant, p <0.010 very significant and p <0.001 is highly significant. Yellow fields indicate significant p-values.

| subtunits |  | GluN1-1a/ 2B | | GluN1-1b/ 2B | |
| --- | --- | --- | --- | --- | --- |
|  |  | without compound | with (*R*)-OF-NB1 | without compound | with (*R*)-OF-NB1 |
| GluN1-1a/ 2B | without compound |  | 0.416 | 0.002 | 0.250 |
|  | with (*R*)-OF-NB1 |  |  | <0.001 | 0.020 |
| GluN1-1b/ 2B | without compound |  |  |  | 0.054 |
|  | with (*R*)-OF-NB1 |  |  |  |  |

Table S5: P-values of open channel probabilities of NMDARs, obtained by single channel recordings of NMDAR containing and without exon 5 (GluN1-1b/ 2B) before in the presence and absence of 1 µM (*R*)-OF-NB1. A p-value <0.050 is significant, p <0.010 very significant and p <0.001 is highly significant. Yellow fields indicate significant p-values.

| subunits |  | GluN1-1a/ 2B | | GluN1-1b/ 2B | |
| --- | --- | --- | --- | --- | --- |
|  |  | without compound | with (*R*)-OF-NB1 | without compound | with (*R*)-OF-NB1 |
| GluN1-1a/ 2B | without compound |  | 0.61 | 1 | 0.932 |
|  | with (*R*)-OF-NB1 |  |  | 0.839 | 0.996 |
| GluN1-1b/ 2B | without compound |  |  |  | 0.988 |
|  | with (*R*)-OF-NB1 |  |  |  |  |


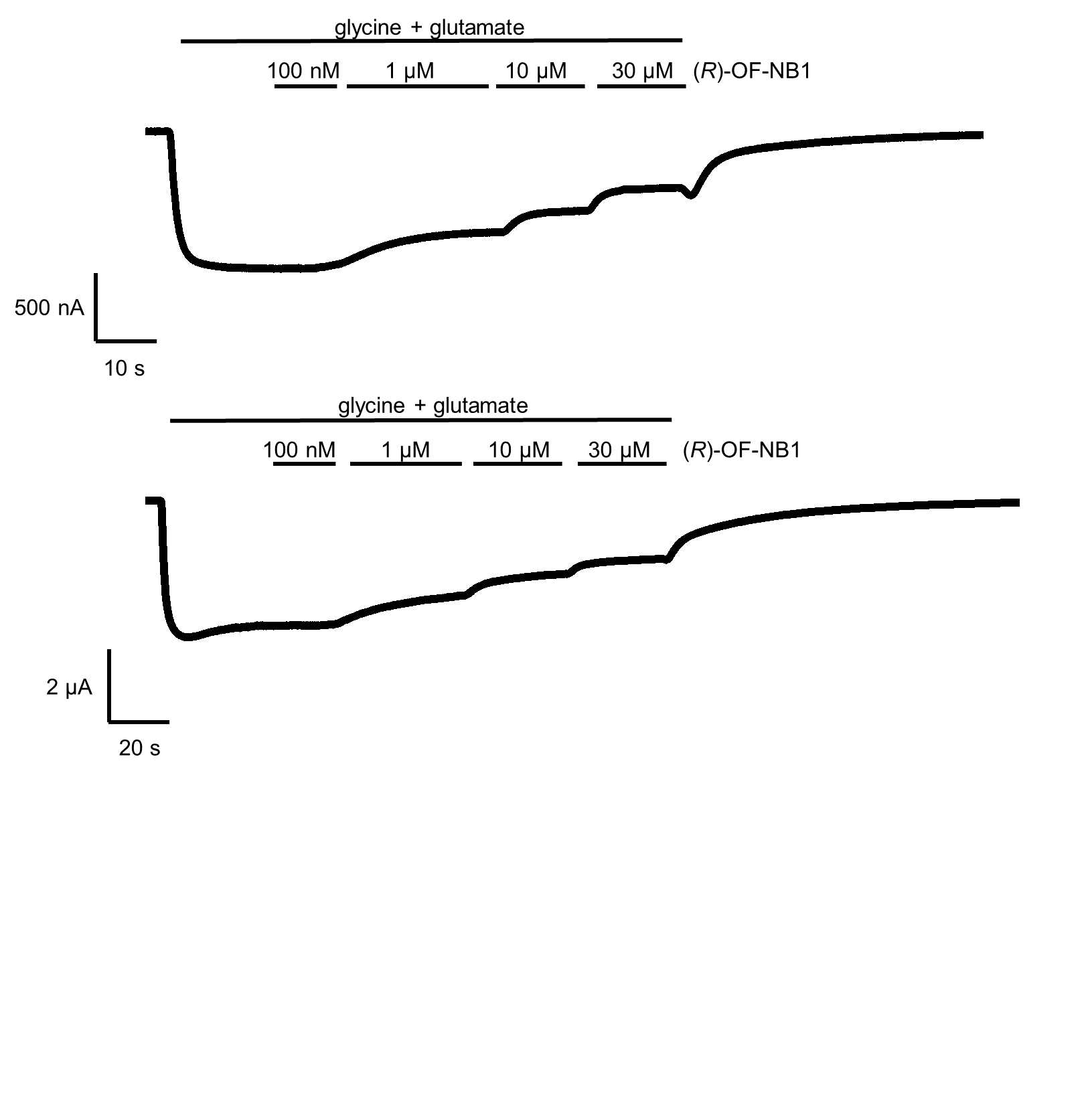


Figure S2: Example traces of TEVC recordings obtained from GluN1-1a wt/ GluN2B F176A (upper) and GluN1-1b wt/ GluN2B F176A (lower) expressing oocytes. NMDARs were first activated by applying 10 µM glycine and 10 µM (*S*)-glutamate, then inhibited by ascending (*R*)-OF-NB1 concentrations (100, 1000, 10000, 30000 nM) in presence of the two agonists. Oocytes were injected with 0.8 ng cRNA of each subunit and recorded after 5 days. Dose response curves were assessed by clamping the oocytes at a holding potential of -70 mV.


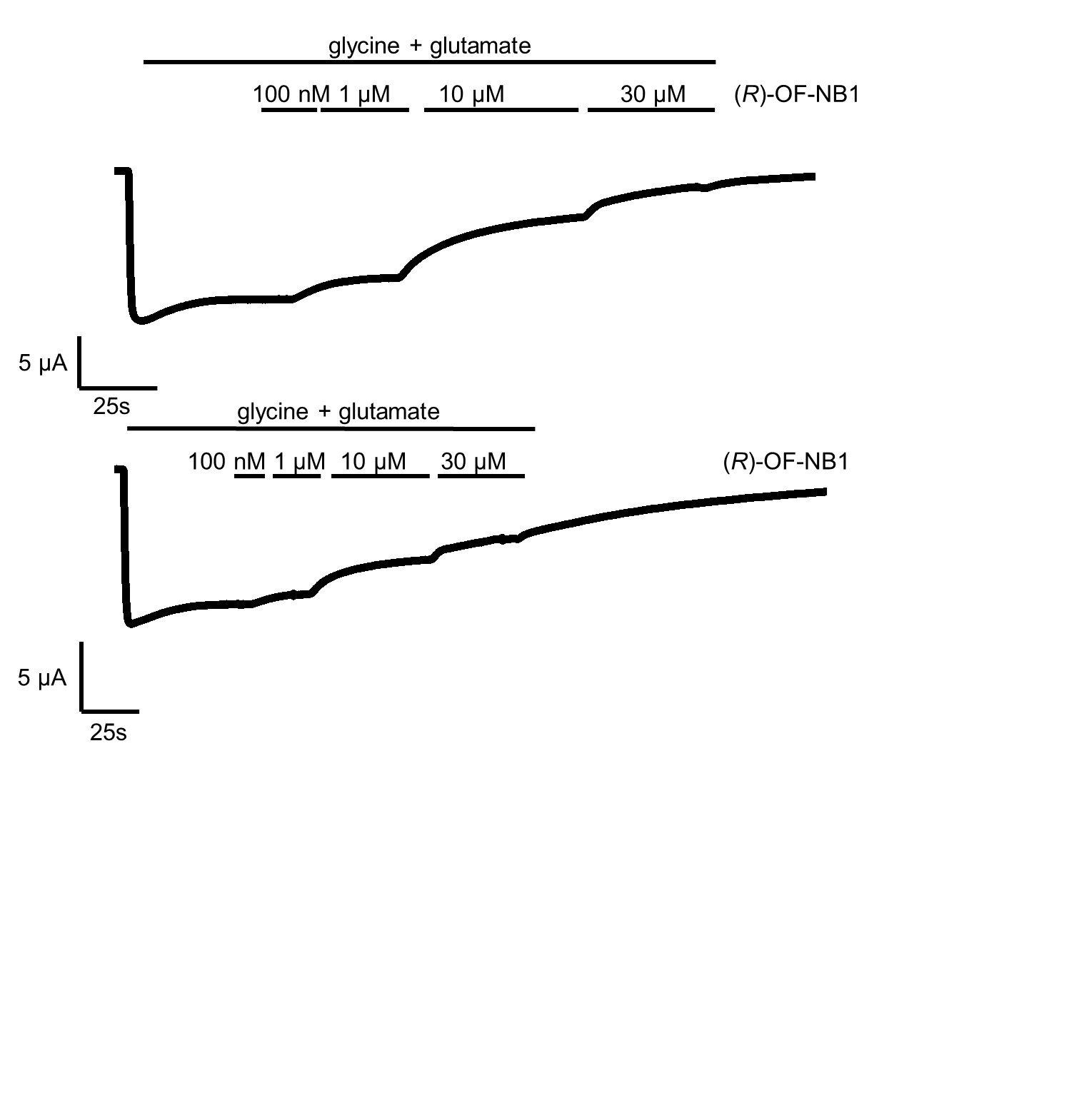


Figure S3: Example traces of TEVC recordings obtained from GluN1-1a wt/ GluN2B F114A (upper) and GluN1-1b wt/ GluN2B F114A (lower) expressing oocytes. NMDARs were first activated by applying 10 µM glycine and 10 µM (*S*)-glutamate, then inhibited by ascending (*R*)-OF-NB1 concentrations (100, 1000, 10000, 30000 nM) in presence of the two agonists. Oocytes were injected with 0.8 ng cRNA of each subunit and recorded after 5 days. Dose response curves were assessed by clamping the oocytes at a holding potential of -70 mV.


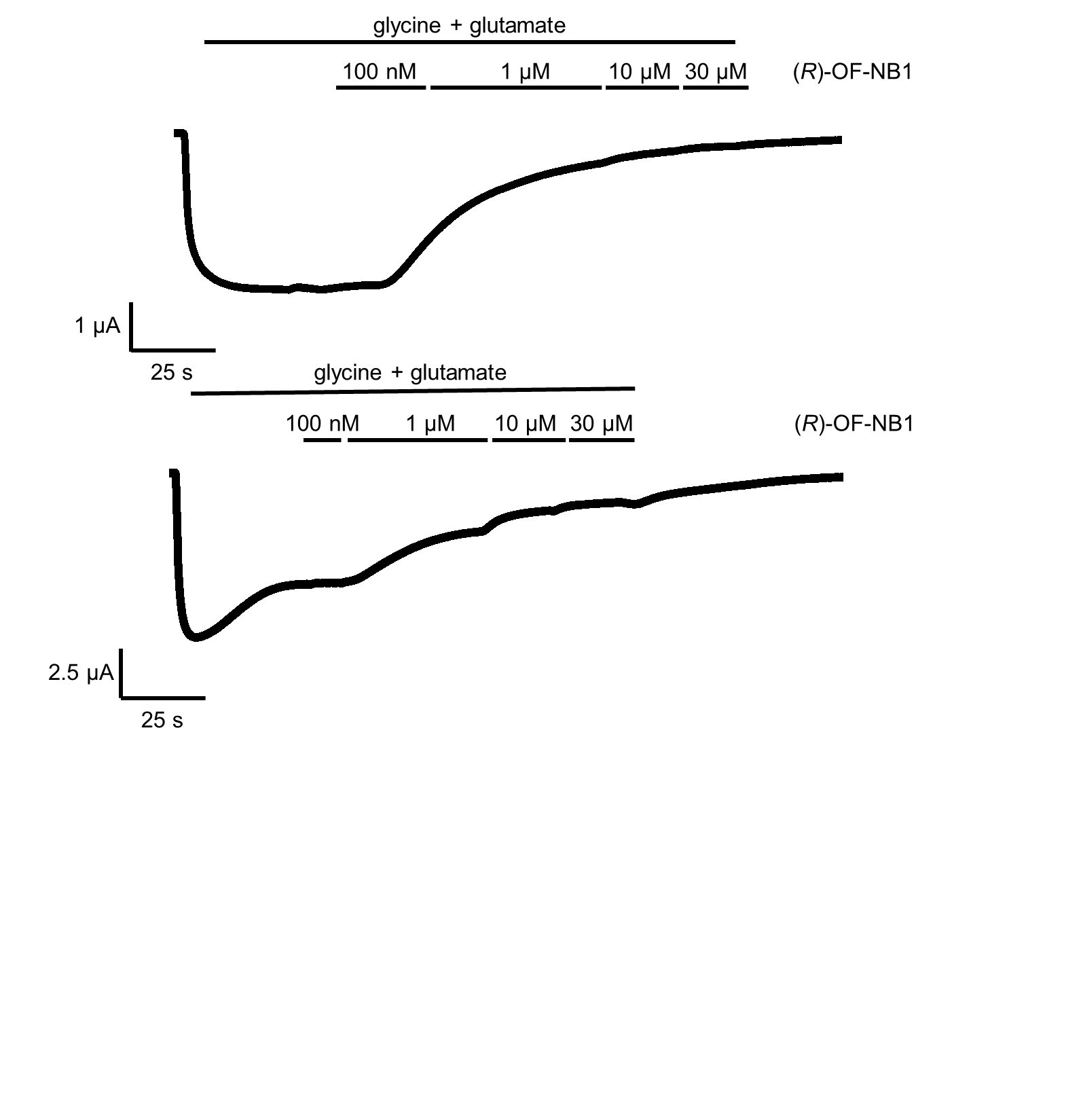


Figure S4: Example traces of TEVC recordings obtained from GluN1-1a wt/ GluN2B Q110A (upper) and GluN1-1b wt/ GluN2B Q110A (lower) expressing oocytes. NMDARs were first activated by applying 10 µM glycine and 10 µM (*S*)-glutamate, then inhibited by ascending (*R*)-OF-NB1 concentrations (100, 1000, 10000, 30000 nM) in presence of the two agonists. Oocytes were injected with 0.8 ng cRNA of each subunit and recorded after 5 days. Dose response curves were assessed by clamping the oocytes at a holding potential of -70 mV.


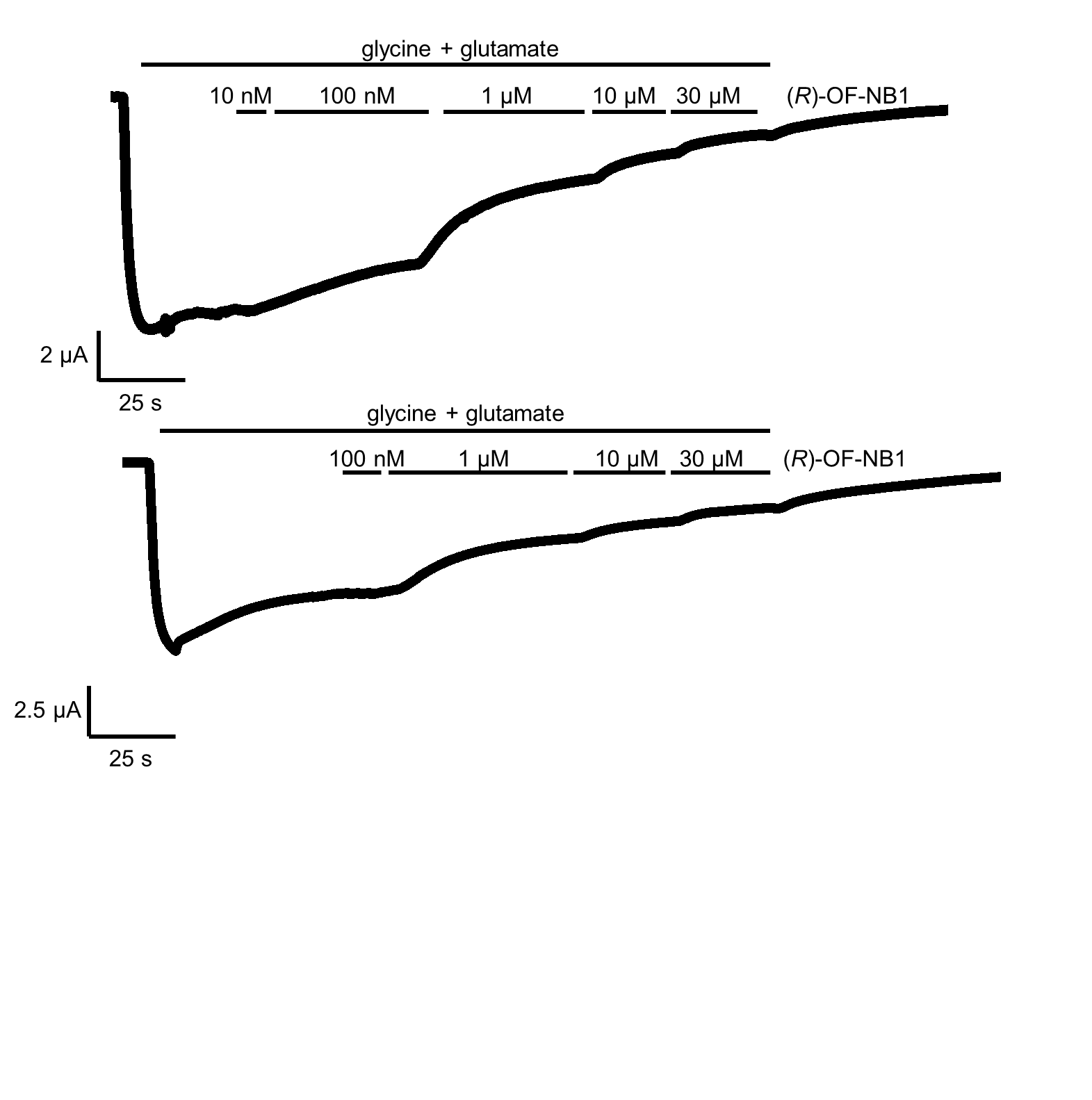


Figure S5: Example traces of TEVC recordings obtained from GluN1-1a wt/ GluN2B E236A (upper) and GluN1-1b wt/ GluN2B E236A (lower) expressing oocytes. NMDARs were first activated by applying 10 µM glycine and 10 µM (*S*)-glutamate, then inhibited by ascending (*R*)-OF-NB1 concentrations (100, 1000, 10000, 30000 nM) in presence of the two agonists. Oocytes were injected with 0.8 ng cRNA of each subunit and recorded after 5 days. Dose response curves were assessed by clamping the oocytes at a holding potential of -70 mV.


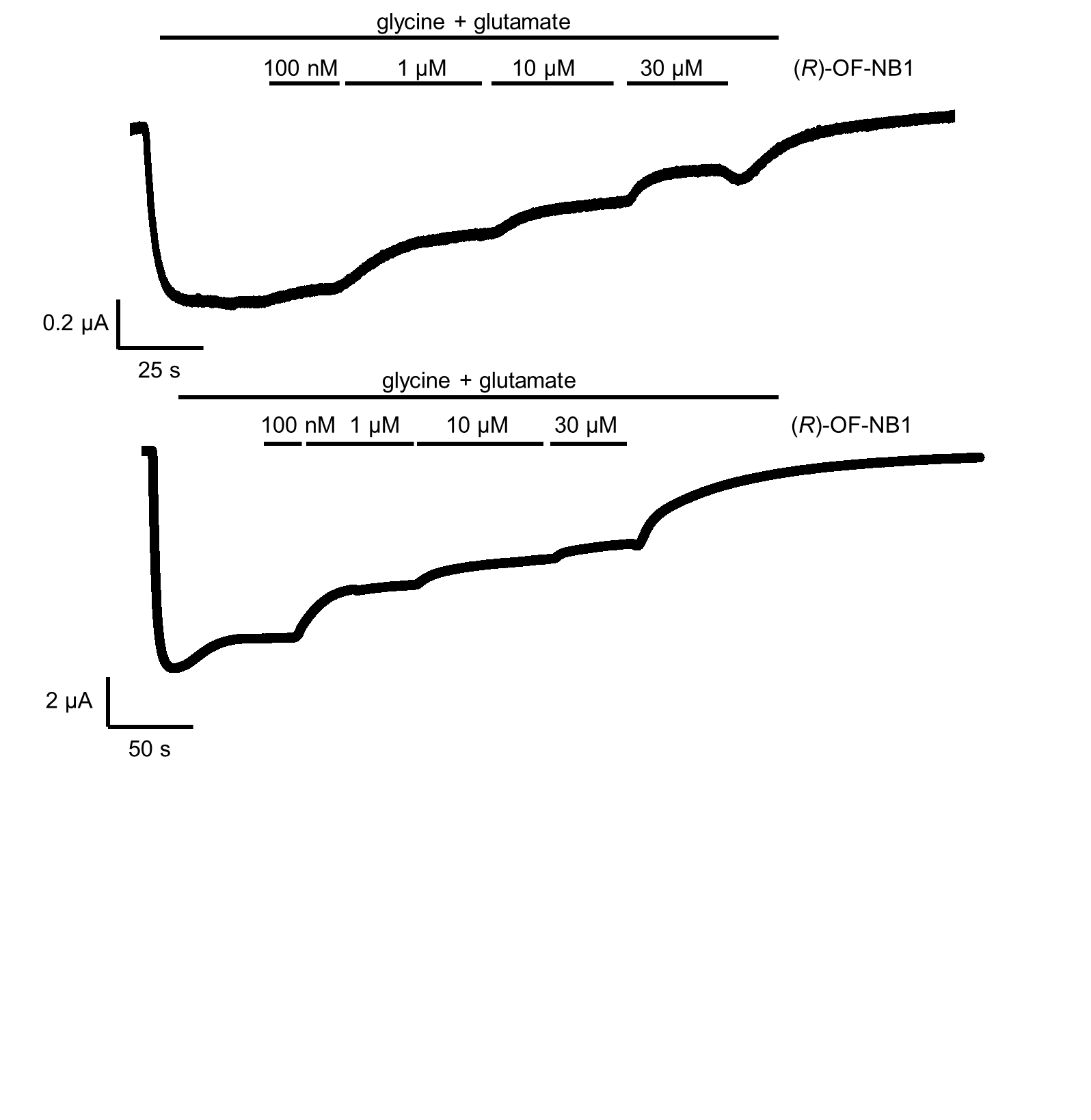
 Figure S6: Example traces of TEVC recordings obtained from GluN1-1a wt/ GluN2B F194A (upper) and GluN1-1b wt/ GluN2B F194A (lower) expressing oocytes. NMDARs were first activated by applying 10 µM glycine and 10 µM (*S*)-glutamate, then inhibited by ascending (*R*)-OF-NB1 concentrations (100, 1000, 10000, 30000 nM) in presence of the two agonists. Oocytes were injected with 0.8 ng cRNA of each subunit and recorded after 5 days. Dose response curves were assessed by clamping the oocytes at a holding potential of -70 mV.


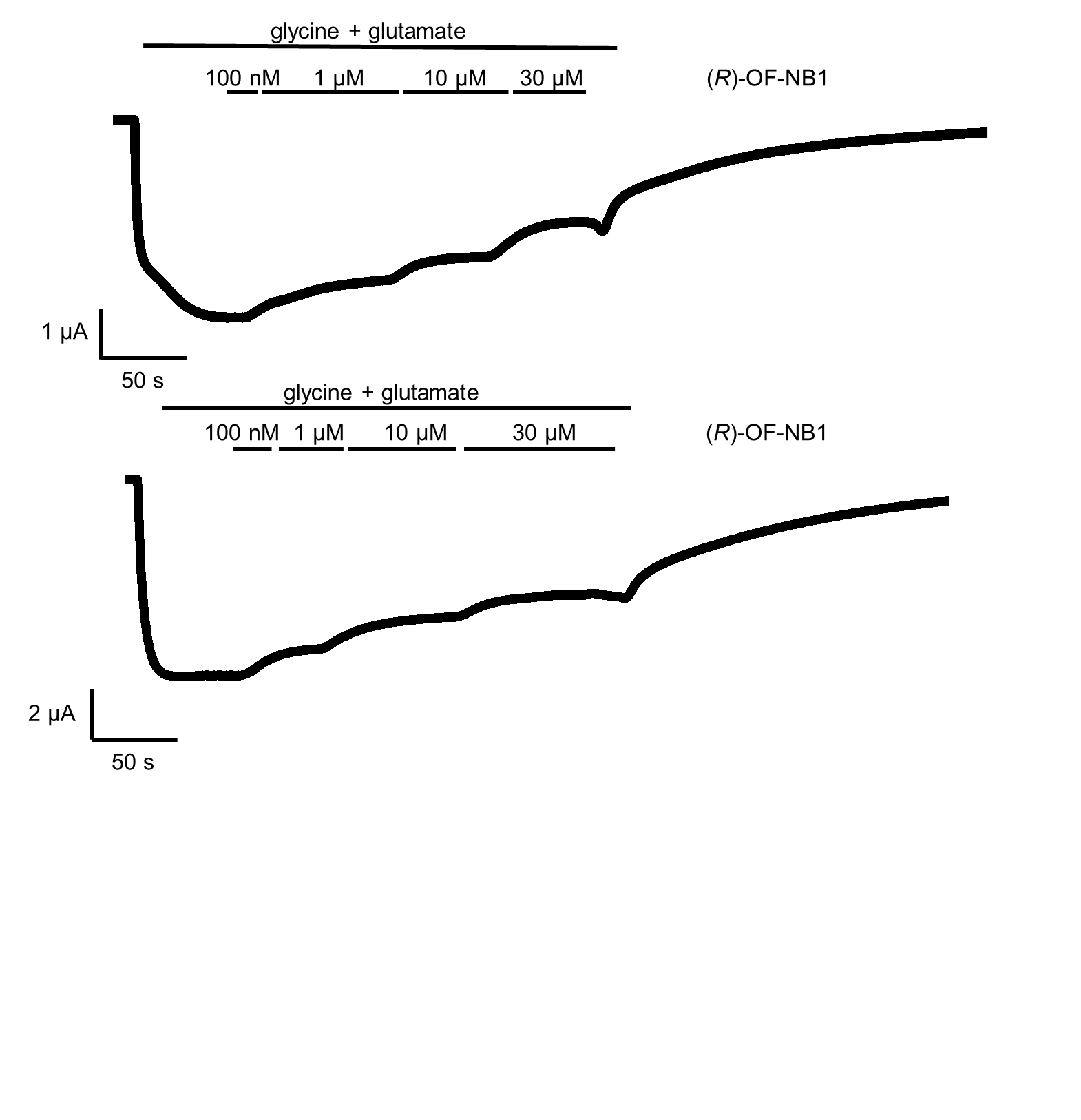


Figure S7: Example traces of TEVC recordings obtained from GluN1-1a wt/ GluN2B N192A/F194A (upper) and GluN1-1b wt/ GluN2B N192A/F194A (lower) expressing oocytes. NMDARs were first activated by applying 10 µM glycine and 10 µM (*S*)-glutamate, then inhibited by ascending (*R*)-OF-NB1 concentrations (100, 1000, 10000, 30000 nM) in presence of the two agonists. Oocytes were injected with 0.8 ng cRNA of each subunit and recorded after 5 days. Dose response curves were assessed by clamping the oocytes at a holding potential of -70 mV.


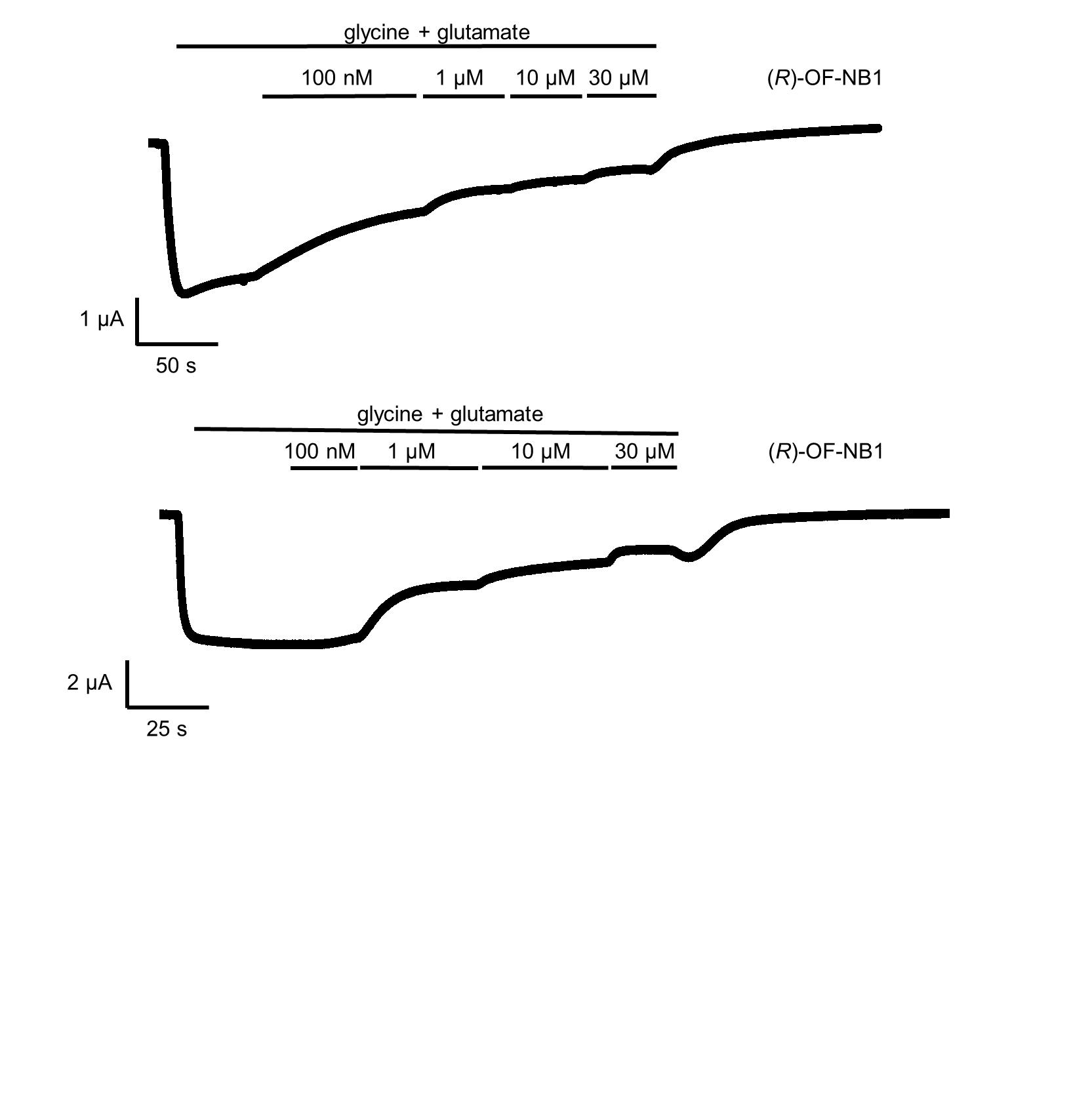
 Figure S8: Example traces of TEVC recordings obtained from GluN1-1a wt/ GluN2B N192A (upper) and GluN1-1b wt/ GluN2B N192A (lower) expressing oocytes. NMDARs were first activated by applying 10 µM glycine and 10 µM (*S*)-glutamate, then inhibited by ascending (*R*)-OF-NB1 concentrations (100, 1000, 10000, 30000 nM) in presence of the two agonists. Oocytes were injected with 0.8 ng cRNA of each subunit and recorded after 5 days. Dose response curves were assessed by clamping the oocytes at a holding potential of -70 mV.


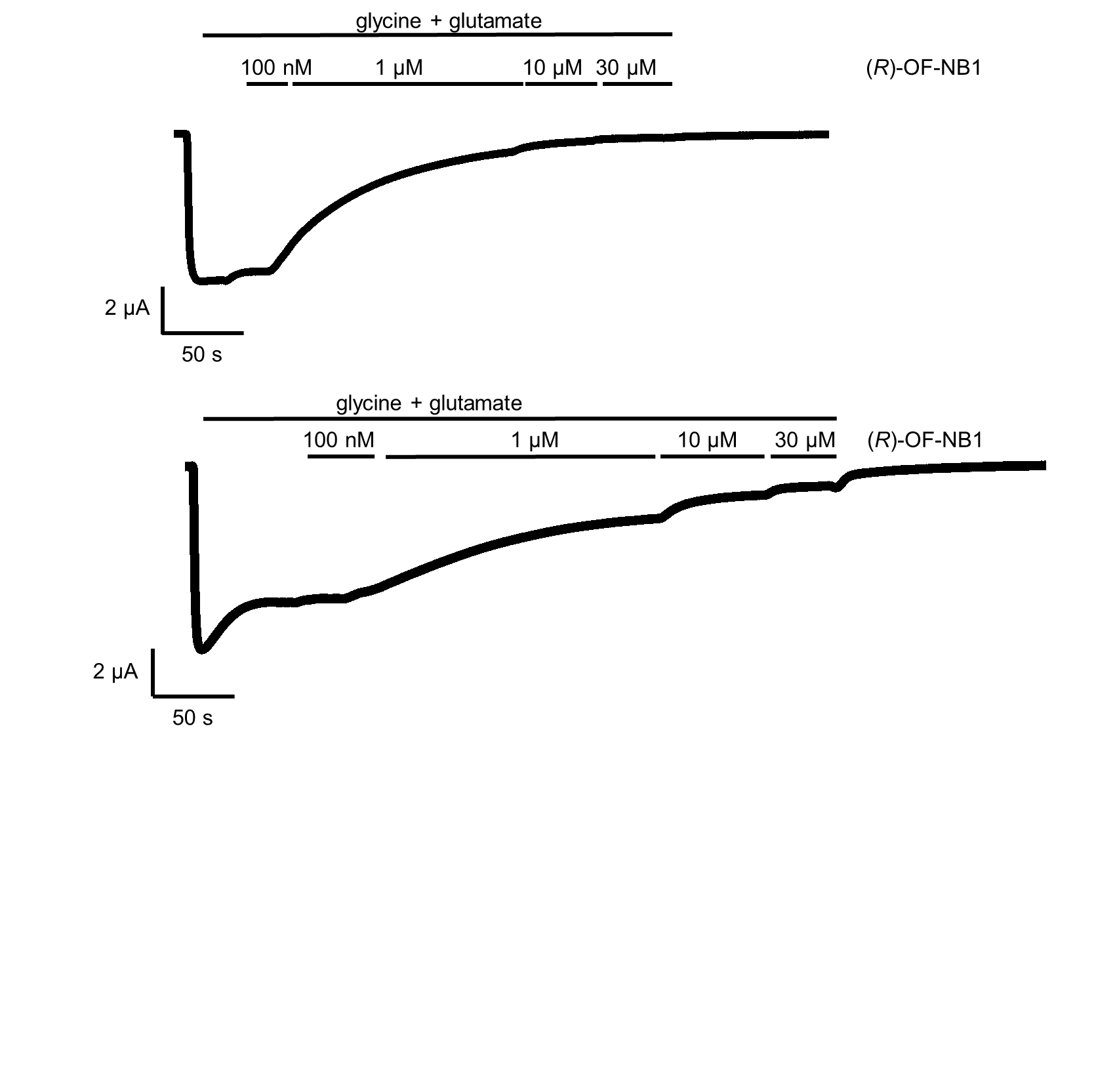
 Figure S9: Example traces of TEVC recordings obtained from GluN1-1a wt/ GluN2B wt (upper) and GluN1-1b wt/ GluN2B wt (lower) expressing oocytes. NMDARs were first activated by applying 10 µM glycine and 10 µM (*S*)-glutamate, then inhibited by ascending (*R*)-OF-NB1 concentrations (100, 1000, 10000, 30000 nM) in presence of 200 µM spermine and the two agonists. Oocytes were injected with 0.8 ng cRNA of each subunit and recorded after 5 days. Dose response curves were assessed by clamping the oocytes at a holding potential of -70 mV.
